# Cholecystokinin Receptor Antagonist Improves Efficacy of Chemotherapy in Murine Models of Pancreatic Cancer by Altering the Tumor Microenvironment

**DOI:** 10.3390/cancers13194949

**Published:** 2021-09-30

**Authors:** Zoe X. Malchiodi, Hong Cao, Martha D. Gay, Anita Safronenka, Sunil Bansal, Robin D. Tucker, Benjamin A. Weinberg, Amrita Cheema, Narayan Shivapurkar, Jill P. Smith

**Affiliations:** 1 Department of Oncology, Georgetown University, Washington, DC 20057, USA; zxm2@georgetown.edu (Z.X.M.); sb1886@georgetown.edu (S.B.); akc27@georgetown.edu (A.C.); 2 Department of Medicine, Georgetown University, Washington, DC 20057, USA; hc87@georgetown.edu (H.C.); mdg111@georgetown.edu (M.D.G.); as4447@georgetown.edu (A.S.); benjamin.a.weinberg@gunet.georgetown.edu (B.A.W.); 3 Department of Pathology, Georgetown University, Washington, DC 20057, USA; rdt24@georgetown.edu

**Keywords:** metastases, pancreatic cancer, tumor microenvironment, epithelial-to-mesenchymal transition, fibrosis, CCK receptor, chemotherapy

## Abstract

**Simple Summary:**

Our research team has identified the cholecystokinin-B receptor (or CCK-BR) as a novel target for treatment of pancreatic cancer. CCK-BRs are over-expressed in pancreatic cancer and activation stimulates growth in cell culture and in animal models. CCK-BRs are also expressed on pancreatic stellate cells—or the fibroblasts that are responsible for the dense fibrosis in the tumor microenvironment that impedes the penetration of chemotherapy. We show strong evidence that treatment with a CCK receptor antagonist, proglumide, decreases fibrosis in the pancreatic tumor microenvironment and increases the influx of T-cells rendering chemotherapy-resistant cancer, sensitive to gemcitabine. Mice treated with the combination therapy had fewer metastases and greater survival. Tumors from these mice had higher gemcitabine levels and novel differentially expressed genes by RNA-Seq. Proglumide is an older drug that was developed many years ago for peptic ulcer disease but is no longer in use. Repurposing this older drug may improve survival from this recalcitrant malignancy.

**Abstract:**

Pancreatic cancer is resistant to chemotherapy in part due to the dense desmoplastic fibrosis surrounding the tumor, the immunosuppressive cells in the tumor microenvironment (TME), and the early rate of metastases. In this study, we examined the effects of a CCK receptor antagonist, proglumide, alone and in combination with gemcitabine in murine models of pancreatic cancer. Tumor growth rate, metastases, and survival were assessed in mice bearing syngeneic murine or human pancreatic tumors treated with PBS (control), gemcitabine, proglumide, or the combination of gemcitabine and proglumide. Excised tumors were evaluated histologically for fibrosis, immune cells, molecular markers, and uptake of chemotherapy by mass spectroscopy. Peripheral blood was analyzed with a microRNAs biomarker panel associated with fibrosis and oncogenesis. Differentially expressed genes between tumors of mice treated with gemcitabine monotherapy and combination therapy were compared by RNAseq. When given in combination the two compounds exhibited inhibitory effects by decreasing tumor growth rate by 70%, metastases, and prolonging survival. Proglumide monotherapy altered the TME by decreasing fibrosis, increasing intratumoral CD8^+^ T-cells, and decreasing arginase-positive cells, thus rendering the tumor sensitive to chemotherapy. Proglumide altered the expression of genes involved in fibrosis, epithelial–mesenchymal transition, and invasion. CCK-receptor antagonism with proglumide renders pancreatic cancer susceptible to chemotherapy.

## 1. Introduction

In spite of the success in the diagnosis and treatment of other cancers over the years, little improvement has occurred in the survival of pancreatic ductal adenocarcinoma (PDAC) [1], which carries the poorest prognosis of all gastrointestinal malignancies [2]. The reasons for the poor survival rates reported for PDAC include the inability to diagnose this disease at early stages, its propensity to metastasize quickly, and its relative resistance to both standard chemotherapy [3] and immunotherapy regimens [4]. Although pancreatic cancer cells may respond to chemotherapeutic agents in cell culture, many of these drugs are less effective in vivo. One probable explanation for the resistant nature of PDAC in the clinic is the dense fibrotic tumor microenvironment (TME) associated with this malignancy [5]. Pancreatic stellate cells (PSCs) have been identified as the specific cellular source of extracellular matrix (ECM) proteins in the PDAC TME [6]. When PSCs are stimulated, they produce collagen and the resultant dense stroma compresses blood vessels, leading to reduced perfusion that ultimately impedes the delivery of drugs to neoplastic cells and prevents the influx of cytotoxic killer T cells [7].

Gemcitabine (2′,2′-difluorodeoxycytidine; dFdC) combined with nab-paclitaxel [8] or the FOLFIRINOX regimen (folinic acid, fluorouracil, irinotecan, and oxaliplatin) [9] are the current standard first-line therapies for advanced PDAC; however, the median survival with these therapies is still less than one year. Since fibrosis and the immunosuppressive nature of the TME have been the major focus for impaired uptake and lack of efficacy of chemotherapeutic agents in PDAC, investigators have turned their attention to strategies that remodel the TME to render tumors more susceptible to chemotherapy. Investigators used hyaluronidase in an attempt to decrease tumor-associated fibrosis and improve chemotherapy delivery [10]. Unfortunately, PEGylated recombinant human hyaluronidase (PEGPH20) combined with nab-paclitaxel and gemcitabine compared to the chemotherapy alone recently failed in the phase 3 HALO-301 clinical trial, and the PEGPH20 arm was associated with significant increase in toxicity, especially venous thromboembolic events [11]. Others have tried to alter the immune cell signature or immunosuppressive TME of PDAC with compounds that block the CCL2-CCR2 chemokine axis from tumor-associated macrophages (TAMs), but these agents have not improved efficacy over standard of care [12]. Recently another immune/cytokine alternating therapy using PEGylated IL-10 and FOLFOX (SEQUOIA trial) also failed as second-line therapy in PDAC [13]. Therefore, novel therapeutic approaches are needed to treat this disease.

Pancreatic cancer and its TME are very complex, and these clinical investigations have revealed to us that interruption of one component of the TME alone is not adequate to inhibit growth and metastases of this aggressive tumor. The various cells of the TME crosstalk through signaling mechanisms and metabolic pathways [14], and blockade of just one cell type seems to enhance immune escape mechanisms, which makes pancreatic cancer very difficult to treat [15]. We have been studying the role of a G-protein coupled receptor, the cholecystokinin-B receptor (CCK-BR), and its signaling pathways in pancreatic cancer. Two classic CCK receptors, the CCK-A (or CCK1) and CCK-B (or CCK2) receptors [16] have been identified. The CCK-AR has a high affinity for only sulfated CCK and a low affinity for gastrin; however, the CCK-BR has equal binding affinity (K_D_~1 nM) for both CCK and gastrin [16]. A number of CCK receptor-specific antagonists have been developed that have affinity to either the CCK-AR or CCK-BR [17]. Proglumide [18] is a nonselective CCK receptor antagonist with properties that inhibit activity for both the CCK-AR and the CCK-BR. CCK receptors become overexpressed in precancerous pancreatic intraepithelial neoplasia (PanIN) lesions [19] and are markedly overexpressed in cancer [20]. Growth of pancreatic cancer in vitro and in vivo is blocked by CCK receptor antagonists [21,22]. CCK receptors have also been identified on fibroblasts [23] and pancreatic stellate cells (PSCs) [24,25]. When the CCK receptors on these cells are stimulated, the cells become activated to produce desmoplastic stroma characteristic of the TME in pancreatic cancer [6,26]. Using the mutant KRAS transgenic mouse model (Pdx1-Cre/LSL-Kras^G12D^) [19], we showed that proglumide-treated mice exhibited regression and failure of PanIN lesions to progress to cancer, when compared to mice treated with vehicle control. Furthermore, mice treated with proglumide had significantly less fibrosis in the TME attributed to blockade of the CCK-receptor on the PSCs. Since CCK receptors are overexpressed and involved with PDAC growth and expressed on fibroblasts of the TME, we hypothesized that CCK receptor blockade would improve the efficacy of gemcitabine in PDAC by modifying the pro-tumorigenic characteristics within the TME.

## 2. Materials and Methods

### 2.1. Cell Lines and Animal Models

Murine pancreatic cancer cells, mT3-2D (mT3), were obtained from Dr. David Tuveson’s laboratory (Cold Spring Harbor, NY, USA) [27]. These cells are syngeneic to C57BL/6 mice; therefore, they can be used in immunocompetent PDAC model(s). The mT3-2D murine tumor recapitulates human PDAC in that it grows rapidly, has mutant Kras^G12D^, and develops the typical dense fibrotic stroma in the TME. PANC-1 human pancreatic cancer cell lines (ATC; Manassas, VA, USA) were used in athymic nude mice. All cells were cultured in DMEM media supplemented with 10% FBS and 1% pen/strep.

C57BL/6 mice and athymic nude mice were purchased from the Charles Rivers (Frederick, MD, USA). All murine studies were performed ethically and approved by the Institutional Animal Care and Use Committee (IACUC) at Georgetown University.

### 2.2. Study Design

Two different animal models were used. In the first model (Supplementary Materials Figure S1A), 500,000 mT3-2D cells were injected subcutaneously into the right flank of 40 female C57BL/6 mice. Tumors were measured weekly using a caliper and the volumes were calculated (L × W^2^ × 0.5). Mice were euthanized per the IACUC protocol when the tumor diameter reached 20 mm. In the second model (Supplementary Material Figure S1B), 800,000 PANC-1 cells that were luciferase transfected were injected orthotopically into the pancreas of 40 male athymic nude mice. Tumor size was monitored every two weeks by measuring luciferase epifluorescence (after 10 mg/mL of luciferin IP) using an IVIS Lumina III in Vivo Optical Imaging System (Perkin Elmer, Waltham, MA, USA). For both experimental models, one week after the mice had measurable tumors (baseline), mice were divided into 4 groups (*n* = 10 mice each) with equal mean tumor volumes. Treatment groups included: PBS control (100 µL IP twice weekly); proglumide in drinking water (0.1 mg/mL); gemcitabine (100 mg/kg, 100 µL IP twice weekly); and combination of gemcitabine (100 mg/kg, 100 µL IP twice weekly) and proglumide in drinking water (0.1 mg/mL).

### 2.3. Tissue Analysis by Histology and Immunohistochemistry (IHC)

Paraffin-embedded tumor sections (5 µm) from all treatment groups were mounted on slides. Metastases were all confirmed by hematoxylin & eosin (H&E) staining. Slides were reacted with Masson’s trichrome stain to assess fibrosis and images were taken on an Olympus BX61 microscope with a DP73 camera (*n* = 10 per slide). Tissue sections were incubated with primary antibodies for CD8^+^ T-cells (Cell Signaling Technology, Danvers, MA, USA, Cat #98941; 1:25) overnight at 4 °C. Slides were then exposed to the appropriate horseradish peroxidase (HRP) labeled polymer for 30 min and diaminobenzidine (DAB) chromagen (Dako) for 5 min. Slides were counterstained with hematoxylin (Sigma-Aldrich, St. Louis, MO, USA), blued in 1% ammonium hydroxide, dehydrated, and mounted with Acrymount. Arginase-positive cells were identified with reacting tissue sections with an arginase antibody (ThermoFisher, Waltham, MA, USA, Cat #PA5-29645) at a dilution 1:1800 for 1 h at room temperature. Slides were exposed to the appropriate HRP-labeled polymer for 30 min. The antibody reaction was detected using DAB as chromogen. Sections were counterstained with hematoxylin to assess arginase-positive cells. IHC images were captured using a 10× objective lens on an Olympus BX61 microscope with a DP73 camera (*n* = 10 per group). Analysis of fibrosis and arginase expression was completed using software by ImageJ. CD8^+^ T cells were counted by calculating the average counts in 4 high powered fields (HPF).

### 2.4. Western Immunoblotting

Tumor tissue was homogenized in RIPA buffer (ThermoFisher) supplemented with PierceTM Protease Inhibitor Tablets, EDTA-Free (ThermoFisher), and protein was isolated. Protein (40 µg) was loaded per sample to assess the expression of fibroblast activated protein (FAP). All protein samples were run on NuPAGE 4–12% Bis-Tris gels (Invitrogen, Waltham, MA, USA). Gels were transferred onto nitrocellulose membranes overnight, semi-dry, at 5 V. Blots were blocked for 30 min with 5% milk (in 1× TBST), then incubated overnight at 4 °C with primary antibody FAP (1:1000; Abcam, Cambridge, UK, ab53066) in 2.5% BSA, then at room temperature for one hour with a rabbit secondary HRP-linked antibody (1:1000; ThermoScientific, Waltham, MA, USA, Cat #31430). For phospho-paxillin, the blot was incubated overnight at 4 °C with primary antibody phospho-paxillin (Tyr118) rabbit polyclonal antibody (1:1000 dilution; ThermoFisher, Cat #44-722G) in 2.5% BSA in PBS, then probed at room temperature for one hour with a goat anti-rabbit secondary HRP-linked antibody (1:1000 dilution in 2.5% BSA in TBST solution; ThermoScientific). For β-catenin, the blot was probed over night at 4 °C with a primary monoclonal murine antibody to β-Catenin (BD Transduction Laboratories; Franklin Lakes, NJ, USA, Cat. #610154, Lot: 25190) at a dilution of 1:2000. This blot was then reacted with HRP-conjugated goat anti-mouse secondary antibody (1:1000 dilution; ThermoScientific; Cat #31430) for one hour at room temperature. The membranes were stripped and probed with β-actin antibody (1:5000, MA1-140; ThermoScientific) overnight at 4 °C followed by the secondary HRP-antibody (1:1000; ThermoScientific, Cat #31430) for one hour at room temperature. Band for FAP, phosphor-paxillin, β-catenin, and β-actin bands were quantified using the open access Image-J software (NIH, Bethesda, MD, USA).

### 2.5. RNA Isolation and Analysis by Quantitative Reverse Transcription PCR (q-RT-PCR) and RNA Sequencing (RNAseq)

Tumor tissue was homogenized in QIAzol Lysis reagent (Qiagen, Germantown, MD, USA) solution then RNA was isolated using miRNeasy mini kit (Qiagen). Synthesis of cDNA was performed using a qScript cDNA Synthesis Kit (Quanta Biosciences, Gaithersburg, MD, USA). Real-time PCR was performed using a PerfeCTa SYBR Green FastMix ROX kit (Quanta Biosciences, Gaithersburg, MD, USA) with an Applied Biosystems 7300 Real Time PCR System machine to assess the expression of Vimentin, Zeb1, Zeb2, SNAIL, β-CATENIN, and TGFβR2. Samples were run for 40 cycles with an annealing temperature of 60 °C. HPRT served as the normalizer control. The primer and normalizer sequences are provided in Supplementary Materials Table S1.

For RNAseq, RNA integrity and quantitation were assessed using the RNA Nano 6000 Assay Kit of the Bioanalyzer 2100 system (Agilent Technologies, Santa Clara, CA, USA) and RIN values > 7.0 were used. Library preparation for transcriptome sequencing was performed by Novogene Co., Ltd. with 1 µg RNA per sample. PCR was performed with Phusion High-Fidelity DNA polymerase, Universal PCR primers and Index (X) Primer. Then, PCR products were purified (AMPure XP system, Beckman Coulter, Brea, CA, USA). Raw data (raw reads) of FASTQ format were firstly processed through fastp. Paired-end clean reads were aligned to the reference genome using the Spliced Transcripts Alignment Reference (STAR) software. Differential expression analysis between the pancreatic tumors from gemcitabine monotherapy were compared to tumors of mice receiving the combination therapy (three biological replicates per condition) was performed using DESeq2 R package. Several of the differentially expressed genes (DEGs) identified were confirmed by PCR.

### 2.6. Mass Spectrometry

A multiple reaction monitoring based Mass Spectrometry method was developed to measure Gemcitabine by UPLC-MS system. The samples were resolved on an Acquity UPLC BEH C18, 1.7 µm, 2.1 × 100 mm^2^ column online with a triple quadrupole mass spectrometer (Xevo-TQ-S, Waters Corporation, Milford, MA, USA). Analysis was performed with a 9-point calibration curve. Gemcitabine showed limit of detection as low as 0.5 pg/mL and was quantifiable in the range 2.5 pg/mL to 1.2 ng/mL. To nullify the matrix effects, calibration curve samples were prepared by spiking standard in blank tissue. Standards were injected twice, at the start and in the end of the batch to check if there is any fall in drug response (due to degradation) during data acquisition. The linearity of drug response in calibration curve points was ascertained by including two quality control (QC) samples in the batch at the start and in the end of the batch. A standard QC and pooled QC samples were injected after every three samples to monitor consistency in the response at a particular drug concentration and ensure minimal instrumental variance. The sample queue was randomized and solvent blanks were injected to assess sample-to-sample carryover. MRM data were processed using Target Lynx 4.1. The relative quantification values of analytes were determined by calculating the ratio of peak areas of transitions of samples normalized to the peak area of the internal standard. A calibration curve for gemcitabine and the representative LC for the drug and internal standard (DBQ) have been included in the Supplementary Materials Figure S2A,B. (See Full Details in Supplementary Methods).

### 2.7. Blood Biomarker miRNA Panel

At euthanasia, murine blood was collected via cardiac puncture and assayed for selective miRNA biomarkers for fibrinolysis: miR-185-5p, miR-346-5p, miR-378a-3p, and miR-708-5p; and for invasion: miR-141, miR-200b, miR-205. Blood serum samples were mixed at a ratio of 1:10 with Qiazol lysis reagent (Qiagen) and vortexed [28,29,30]. The lysate was then extracted with CHCl3 and the aqueous phase was further processed for total RNA using the miRNeasy Mini Kit (Qiagen). miRNA was reverse transcribed to cDNA using miScriptII RT kit (Qiagen). miRNA expression profiling for specific miRNAs was performed using miScript primer assays (Qiagen) and miScript SYBR green PCR kit (Qiagen) on an ABI 7300 Real-Time PCR system (Applied Biosystems, Waltham, MA, USA) following manufacturer’s instructions [29,30]. A dissociation curve analysis of PCR products was carried out to confirm the specificity of amplification. Data were normalized using U6 as endogenous controls. The relative differences between two groups were calculated using ∆∆C_T_ method. Significance between data from two groups will be determined with Prism GraphPad Version 5.03 by using unpaired Student *t*-test (*p* < 0.05).

### 2.8. Statistical Analysis

All statistical analyses were performed using GraphPad Prism software. Tumor growth was calculated by regression analysis and *t*-test between the slopes of the lines. Survival analysis was performed with a Kaplan-Meier Survival Curve of mice. Log-rank analysis was performed comparing controls to each treatment group by applying a Cox proportional hazard model. Differences between groups of PANC-1 final tumor mass were analyzed by two-way ANOVA. The mean number of metastases, number of arginase-positive cells, number of CD8^+^ T-cells and fibrosis density scores per group were compared to PBS controls by paired Student’s *t*-test and Bonferroni correction for multiple comparisons. The blood biomarker miRNA analysis was performed with two-way ANOVA followed by Tukey multiple comparison test was used for the analysis compared to PBS.

## 3. Results

### 3.1. Combination Therapy with Gemcitabine and a CCK Receptor Antagonist Significantly Slows Pancreatic Tumor Growth, Prolongs Survival, and Decreases Metastases

**Figure 1:**
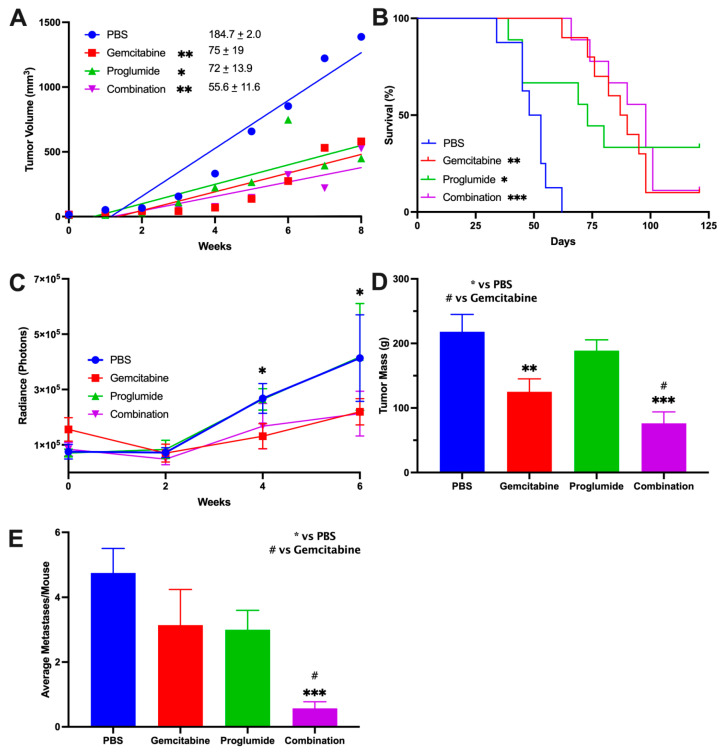
Combination therapy with gemcitabine and a CCK receptor antagonist significantly slows pancreatic tumor growth, prolongs survival, and decreases metastases. (**A**) Tumor volumes for the first eight weeks while all mice were alive are shown for the mT3-2D tumors in C57BL/6 immunocompetent mice. The slope of the growth curve for PBS-treated control mice was the greatest (184.7 ± 2.0). Gemcitabine (slope = 75 ± 19) and proglumide (slope = 72 ± 13.9) growth rates were comparable. The mice treated with a combination of gemcitabine and proglumide had the slowest growth rate (slope = 55.6 ± 11.6). Statistically significant compared to control (* *p* < 0.05, ** *p* < 0.005). (**B**) Kaplan–Meier survival curve of mice in all treatment groups. By day 62, all of the PBS-treated (control) mice died. The median survival was significantly increased for combination therapy, followed by gemcitabine, proglumide, and then the PBS-control group. Log-rank was significant compared to controls (* *p* = 0.01, ** *p* = 0.005, *** *p* < 0.0005). (**C**) Primary tumor size of human PANC-1 orthotopic tumors were measured by luciferase flux and show equal flux at baseline (week 0) when treatment was initiated. Over time, the luciferase flux of the tumors was less in the mice receiving combination therapy with gemcitabine and proglumide compared to PBS controls. By week 6, the tumors of mice receiving gemcitabine monotherapy also exhibited less flux compared to controls (* *p* < 0.05). (**D**) Final PANC-1 tumor mass at the end of the experiment shows significantly smaller tumors in gemcitabine treated mice (* *p* = 0.042) and tumors in the mice receiving combination therapy had even smaller primary tumors (** *p* = 0.0017, *** *p* < 0.0005, ^#^ *p* < 0.05). (**E**) Metastases are decreased by the combination of gemcitabine and proglumide. Columns represent the means ± SEM of the number of metastatic lesions identified and confirmed by histology per mouse in each group. Combination therapy significantly reduce the total number and mean number of metastases per mouse (*** *p* < 0.0005, ^#^ *p* < 0.05).

The mT3-2D murine tumor recapitulates human PDAC in that it grows rapidly, has mutant KRAS^G12D^, and develops the typical dense fibrotic stroma in the TME [27,31]. Using this immunocompetent model, we found that therapy with the CCK receptor antagonist, proglumide, slowed the growth of pancreatic tumors by the same magnitude as gemcitabine treatment compared to PBS-treated controls (Figure 1A). The growth rate, as measured by the mean slope of tumor volumes for proglumide, was decreased by 59.4% when compared to that of PBS-treated control mice (*p* < 0.005). Similarly, the tumor growth rate in gemcitabine-treated mice decreased by 60% compared to that of control mice (*p* < 0.005). These results demonstrate that monotherapy with proglumide is effective in slowing pancreatic cancer growth and is similar to that of gemcitabine. The combination therapy with gemcitabine and proglumide (Figure 1A) resulted in a 70% reduction in tumor mT3-2D pancreatic tumors’ growth (*p* < 0.005) compared to PBS-treated control mice and a further 26% reduced growth rate compared to gemcitabine monotherapy. The reduced growth rates with combination therapy suggest that the two drugs may have an inhibitory effect, and that proglumide improves the efficacy of gemcitabine. The Kaplan–Meier survival curve (Figure 1B) shows that the combination therapy group had significantly greater survival compared to controls calculated by log-rank test (*p* < 0.001). The median survival was best for the combination group followed by gemcitabine, and then proglumide; the PBS control mice exhibited the poorest survival. By day 62, all of the control PBS-treated mice had been euthanized since the tumors reached the maximum allowed size of 20 mm in diameter (Figure 1B). At this same time point (day 62), three mice in the proglumide monotherapy-treatment group had died, one mouse in the gemcitabine-only group had died, and none in the combination group died. On day 87, survival analysis by Kaplan–Meier curve of the other groups was as follows: proglumide 40% (*p* = 0.01); gemcitabine 50% (*p* = 0.001), and combination group 70% (*p* < 0.0001). The survival data demonstrate a significant advantage of utilizing combination therapy in the treatment of pancreatic cancer. There were three complete responses with loss of tumor in the proglumide group, one complete response in the gemcitabine monotherapy group, and one complete response in the group treated with the combination therapy of both gemcitabine and proglumide.

Similar inhibitory effects were observed when proglumide was administered in combination with gemcitabine in the second murine model, using an orthotopic gemcitabine-resistant human pancreatic tumor (PANC-1) in immune deficient mice. One week after tumor inoculation, (week 0, baseline) the luciferase flux was equal in all four treatment groups using the IVIS; (Figure 1C), and therapy was initiated. Tumor size was measured by luciferase flux every 2 weeks up to week 6, and the size of the tumors was less in the combination-therapy mice at weeks 4 and week 6 than that of controls (Figure 1C; *p* < 0.05) and gemcitabine treated mice had less luciferase flux at week 6 when compared to PBS-treated control mice (*p* < 0.05). After 6 weeks, when the mice were ethically euthanized, the tumors of proglumide-treated mice were 14% less in mass compared to PBS controls; and was not significant. Tumors of gemcitabine treated mice weighed 42.5% less than PBS controls (Figure 1D; *p* < 0.05). Tumors of mice treated with the combination therapy were 59% less in mass than PBS treated tumors (*p* = 0.0017); however, these tumors were not significantly different from the mass of the tumors from the gemcitabine-monotherapy treated mice.

One of the most noteworthy findings in this orthotopic model, however, was that the total number of histologically confirmed PANC-1 pancreatic cancer metastases was significantly decreased in the mice with orthotopic tumors treated with the combination of gemcitabine and proglumide (Figure 1E). The mean number of metastases identified per mouse was reduced by 89% in mice treated with the combination therapy compared to PBS-treated control mice (Figure 1E; *p* = 0.0004). The total number of metastases counted in PBS mice were 45, whereas the number of metastases in gemcitabine and proglumide-treated mice were slightly less at *n* = 30 each (Figure 1E). The effect of combining gemcitabine and proglumide was inhibitory on reducing the number of metastases and only *n* = 6 total metastases were counted in this treatment group. The number of metastases according to location is shown in Supplementary Materials Table S2 with the most metastases located in the peritoneal cavity or mesentery. All of the metastases were counted upon dissection and confirmed by histology and representative photos shown in Supplementary Figure S3A–H, which include metastases to the abdominal wall, the liver, the spleen, mesentery, and peritoneum.

These two murine models of pancreatic cancer indicate that when proglumide is administered concomitantly with gemcitabine there is a beneficial effect, with smaller primary tumor size, prolonged survival, and decreased metastases. The beneficial effect observed by combining proglumide and gemcitabine is consistent regardless of the immune status or gender of the animals.

### 3.2. CCK Receptor Blockade with Proglumide Decreases Fibrosis in the Pancreatic TME

**Figure 2:**
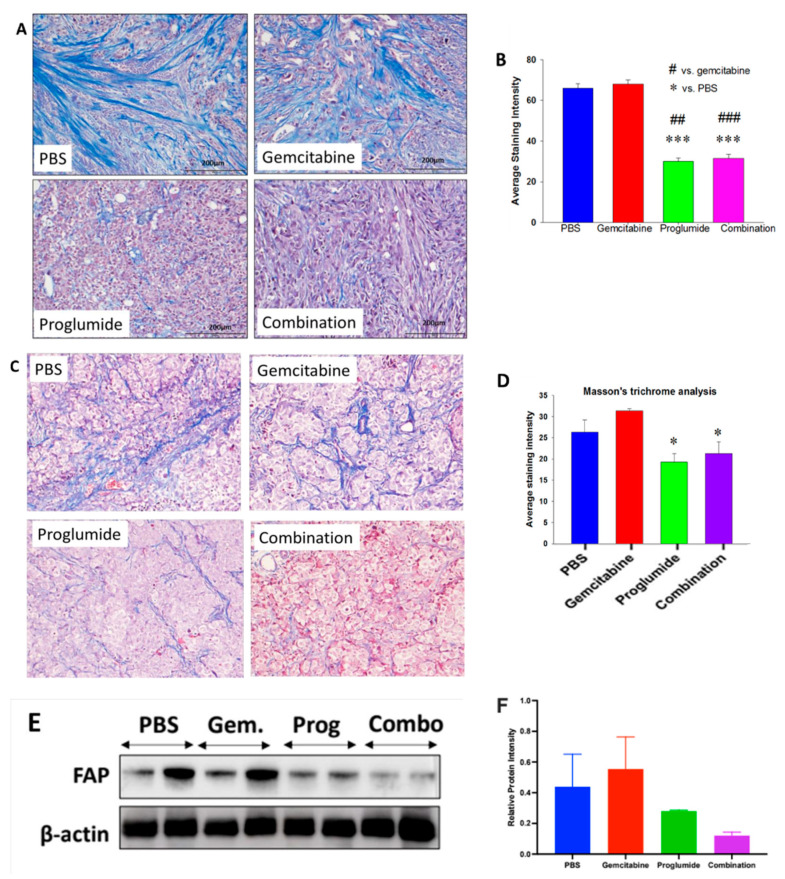
CCK receptor inhibition with proglumide decreases fibrosis in the pancreatic TME. (**A**) Representative images from each treatment group of tumors from the mT3-2D subcutaneous murine pancreatic cancer reacted with Masson’s trichrome stain to demonstrate fibrosis in the pancreatic tumor microenvironment. Images shown are at magnification 10× (bar = 200 µm). (**B**) Quantitative analysis of fibrosis for the murine syngeneic mT3-2D tumors was determined by ImageJ. Mean ± SEM from *n* > 50 images. Significantly less fibrosis was detected in the proglumide and combination-treated tumors (^#^ compared to gemcitabine, * compared to PBS controls; ^##^ *p* < 0.005, *** *p* < 0.005, ^###^ *p* < 0.001). (**C**) Representative images from each treatment group of PANC-1 orthotopic tumors reacted with Masson’s trichrome stain to demonstrate fibrosis in the pancreatic tumor microenvironment. Images shown are at magnification 10×. (**D**) Quantitative analysis of fibrosis in PANC-1 tumors was determined by ImageJ. Mean ± SEM from *n* ≤ 50 images. Significantly less fibrosis was detected in the proglumide and combination treated tumors (* *p* < 0.05). (**E**) Western blot of protein from (*n* = 2) representative homogenates of the mT3 = 2D tumors examining the protein expression of FAP and normalized with β-actin. (The whole western blot is shown in the Figure S4). (**F**) Western blot quantification of FAP by densitometry and each sample normalized by corresponding β-actin performed on ImageJ, *n* = 2/group.

Pancreatic cancer characteristically develops a dense fibrotic microenvironment surrounding the tumors’ cells that is thought to prevent the permeation of chemotherapy and influx of CD8^+^ T cells. Intratumoral fibrosis was analyzed by Masson’s trichrome stain; and a representative image of fibrosis in the mT3-2D tumors from each treatment group is shown in Figure 2A. The intensity of the staining is most intense in the PBS-treated control tumors (Figure 2A). Intratumoral fibrosis was visibly decreased in the tumors of mice receiving proglumide either as monotherapy or in combination with gemcitabine. Quantitative morphometric densitometry analysis revealed that intratumoral fibrosis was significantly decreased in proglumide-treated mice (Figure 2B; *p* < 0.0001) compared to PBS-treated or gemcitabine monotherapy-treated mice. Gemcitabine treatment exhibited no decrease in tumoral fibrosis.

PANC-1 tumors grown orthotopically in immune deficient mice also showed intratumoral fibrosis (Figure 2C), but the staining was not as intense as the mT3-2D tumors from the immune competent mice. Quantification of the fibrosis showed that gemcitabine therapy did not decrease fibrosis in the TME (Figure 2D) compared to PBS-treated control mice bearing PANC-1 tumors. Similar to the mT3-2D pancreatic tumors grown in immune competent mice, PANC-1 orthotopic tumors from mice treated with proglumide monotherapy or combination proglumide therapy with gemcitabine also showed decreased fibrosis compared to PBS-treated controls and also compared to gemcitabine alone (Figure 2D). The marked decrease in tumoral fibrosis observed in both murine models is only seen in the mice treated with proglumide implying that the anti-fibrotic effect is attributed to proglumide.

Fibroblast activation protein (FAP) is generally highly expressed in PDAC tumors; therefore, we also measured FAP protein expression by Western blot in the mT3-2D subcutaneous tumors (Figure 2E). Proglumide monotherapy and its use in combination with gemcitabine exhibited decreased FAP expression when evaluated by densitometry and normalized to β-actin (Figure 2F), suggesting that FAP-positive fibroblasts are inhibited by proglumide treatment.

### 3.3. Proglumide Therapy Alters the Tumor Immune Cell Signature

**Figure 3:**
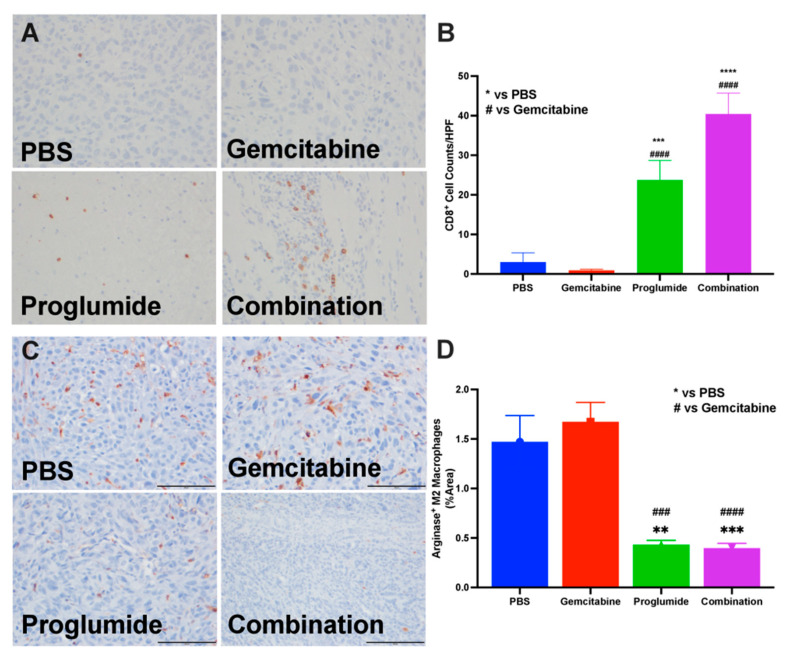
Proglumide therapy alters the tumor immune cell signature. (**A**) A representative tumor section from each treatment group showing immunoreactivity for CD8^+^ tumor-infiltrating lymphocytes from mT3-2D tumors. The 5 µm tissue sections were imaged with a 10× objective, 200 µm. (**B**) Analysis of the number of tumor-infiltrating CD8^+^ T-lymphocytes is shown. Columns represent means per group ± SEM (^#^ compared to gemcitabine, * compared to PBS controls; *** and ^###^ *p* < 0.001; **** and ^####^ *p* < 0.0001). (**C**) Representative images from tumors of each treatment group of the mT3-2D tumors shows immunoreactivity for arginase indicating immune suppression within the TME in each tumor section. (**D**) Analysis of the percentage of arginase positive cells per area examined on each tumor section. Columns represent means per group ± SEM (^#^ compared to gemcitabine, * compared to PBS controls; ** *p* < 0.005; *** and ^###^ *p* < 0.001; ^####^ *p* < 0.0001).

Tumor-infiltrating lymphocytes (TILs) in the pancreatic TME, particularly T cells, have been positively associated with patient prognosis [32]. Tumor sections from all the groups in the syngeneic mT3-2D tumors of immunocompetent mice were stained for CD8^+^ T lymphocytes by immunohistochemistry (IHC) and images scanned by microscope as described above. Representative tumor images from PBS and gemcitabine-treated mice had a paucity of CD8^+^ TILs (Figure 3A, top). In contrast, CD8^+^ T cells were visibly increased in the tumors of all the mice treated with proglumide (Figure 3A, bottom). The number of CD8^+^ T lymphocytes per slide was counted and mean values from each group revealed a statistically increased number of T cells in the tumors of mice treated with proglumide (Figure 3B; *p* < 0.0001).

Another feature of the pancreatic TME is the abundance of arginase expression, indicative of a more immunosuppressive TME. IHC staining of tumor sections from PBS- or gemcitabine-treated immune competent mice had an abundance of arginase-positive cells within the TME (Figure 3C, top). In contrast, arginase-positive cells were sparse within the TME of mice treated with proglumide monotherapy or proglumide in combination with gemcitabine (Figure 3C, bottom). The number of arginase-positive cells was counted, and the mean numbers were statistically greater in the tumors of both the PBS- and gemcitabine-treated mice (Figure 3D). Tumors of the mice treated with proglumide had 73% fewer arginase-positive cells in the TME (Figure 3D; *p* < 0.001). The increase in TILs and decrease in arginase-positive cells within the proglumide-treated groups rendered the TME more immune responsive.

### 3.4. Combination Therapy with Gemcitabine and Proglumide Decreases Metastases by Preventing Epithelial-to-Mesenchymal Transition (EMT)

**Figure 4:**
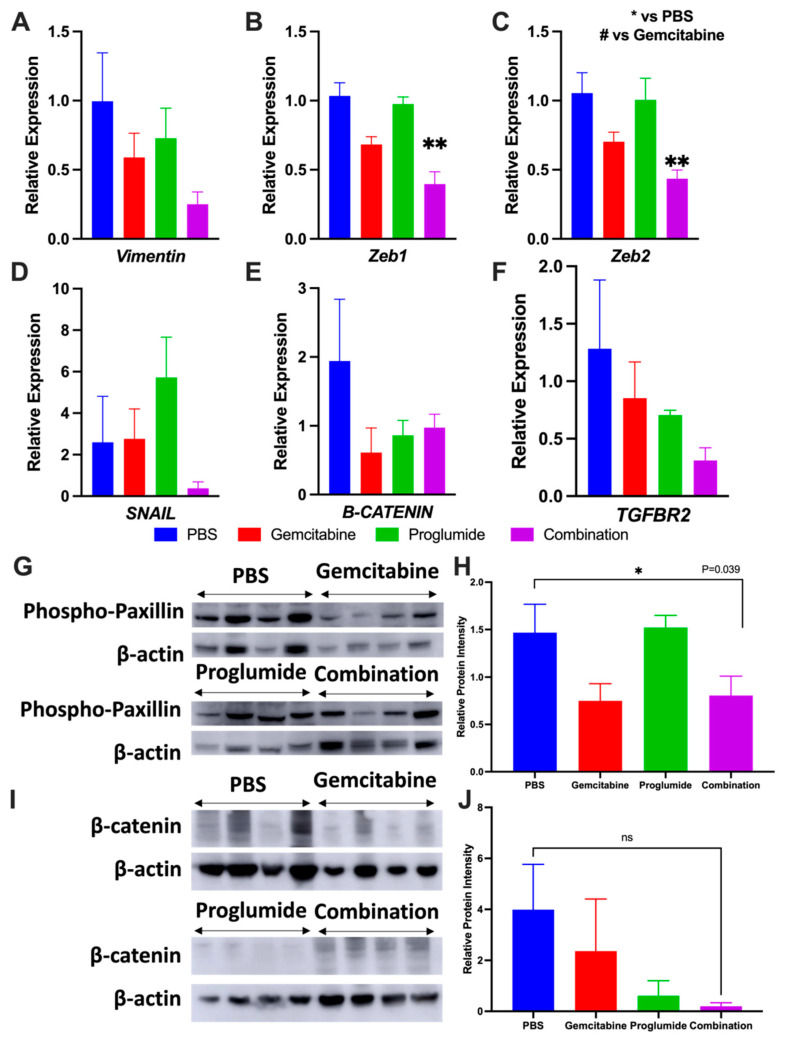
Combination therapy with gemcitabine and proglumide decreases metastases by preventing epithelial to mesenchymal transition (EMT). Quantitative PCR analysis for mRNA expression of transcription factors and proteins from PDAC tumors (*n* = 4 per group) with relative gene expression (2^−ΔΔCt^ ± SEM) for: (**A**) *Vimentin*, (**B**) *Zeb1*, (**C**) *Zeb2*, (**D**) *SNAIL1*, (**E**) *β-CATENIN*, and (**F**) *TBFβR2*. Unpaired t-test was used for analysis (* *p* = 0.05; ** *p* < 0.01) and *GAPDH* was used as the loading control. (**G**) Western blot for phospho-paxillin for tumor protein extracts of each group normalized with *β*-actin. (The whole western blot is shown in the Figure S4). (**H**) Analysis of bands by densitometry show significant differences in the groups * *p* < 0.05. (**I**) Western blot of PANC-1 tumor protein extracts reacted with an antibody for β-catenin normalized with *β*-actin. (The whole western blot is shown in the Figure S4). (**J**) Analysis of the bands by densitometry show a decrease in the intensity of the bands in the proglumide and combination groups although this did not quite reach statistical significance.

Cancer invasion and metastasis are preceded by a phenotypic transformation in cancer cells through a process called epithelial-to-mesenchymal transition (EMT) [33] that is regulated by a network of cytokines, transcription factors, growth factors and signaling pathways in the TME [34]. Vimentin is a constituent of the intermediate filament family of proteins expressed in mesenchymal cells and a canonical marker of EMT [35]. Increased vimentin expression in PDAC has been associated with a poor outcome with shorter survival [36]. Vimentin mRNA expression by qRT-PCR was high in tumors of control mice treated with PBS (Figure 4A) and was significantly decreased in tumors of mice treated with the combination of proglumide and gemcitabine. Zeb1 or Zinc finger E-box binding homeobox 1 (ZEB1) is a transcription factor involved in EMT and functions as a repressor of the tumor suppressor protein E-cadherin [37]. Zeb1 expression was markedly suppressed in tumors of mice treated with the combination therapy (Figure 4B) compared to PBS or monotherapy with proglumide or gemcitabine. Zeb2 is another zinc finger transcription factor that is increased in EMT and functions as a DNA-binding transcriptional repressor that interacts with activated SMADs [38]. Similarly, Zeb2 expression was significantly decreased in tumors of mice treated with the combination therapy compared to control mouse tumors (Figure 4C).

Expression of the transcription factor SNAIL is also increased in PDAC, and increased levels are associated with advanced disease and metastasis [39]. Expression of SNAIL was decreased in PANC-1 tumors of mice treated with the combination of gemcitabine and proglumide (Figure 4D). High expression of β-catenin is also associated with EMT in many tumor types [40]. PANC-1 tumors from mice treated with the combination of gemcitabine and proglumide had decreased expression of β-catenin compared to that of the tumors of control mice (Figure 4E). EMT can be induced by the activation of the TGFβ signaling pathway to promote metastasis [41] and recently signaling and upregulation of the TGFβ2 receptor (TGFβR2) has been associated with cancer invasiveness and increased metastasis [42]. mRNA expression of TGFβR2 was found to be downregulated in tumors of mice receiving the combination therapy that exhibited fewer metastases (Figure 4F). Western blots of PANC-1 tumor protein extracts that were reacted with an antibody to phospho-paxillin were decreased in tumors of mice treated with the combination therapy (Figure 4G,H). Gastrin has been shown to promote the reorientation of the Golgi apparatus and directional migration of pancreatic cancer cells by inducing the activation of paxillin [43]. Paxillin is a focal adhesion protein that when activated promotes cell motility and migration. Similarly, β-catenin protein expression was also decreased in tumors of mice treated with the combination therapy (Figure 4I,J); however, this difference did not quite reach significance. β-catenin is involved in cell–cell adhesion through interaction with the E-cadherin cell-adhesion complex [44], and gastrin has been shown to increase metastases by inducing β-catenin nuclear translocation [45]. These results support that evidence that the therapy with proglumide and gemcitabine in combination decreased metastases by downregulating proteins and transcription factors of the EMT pathway.

### 3.5. Analysis of Tumors by RNAseq

**Figure 5:**
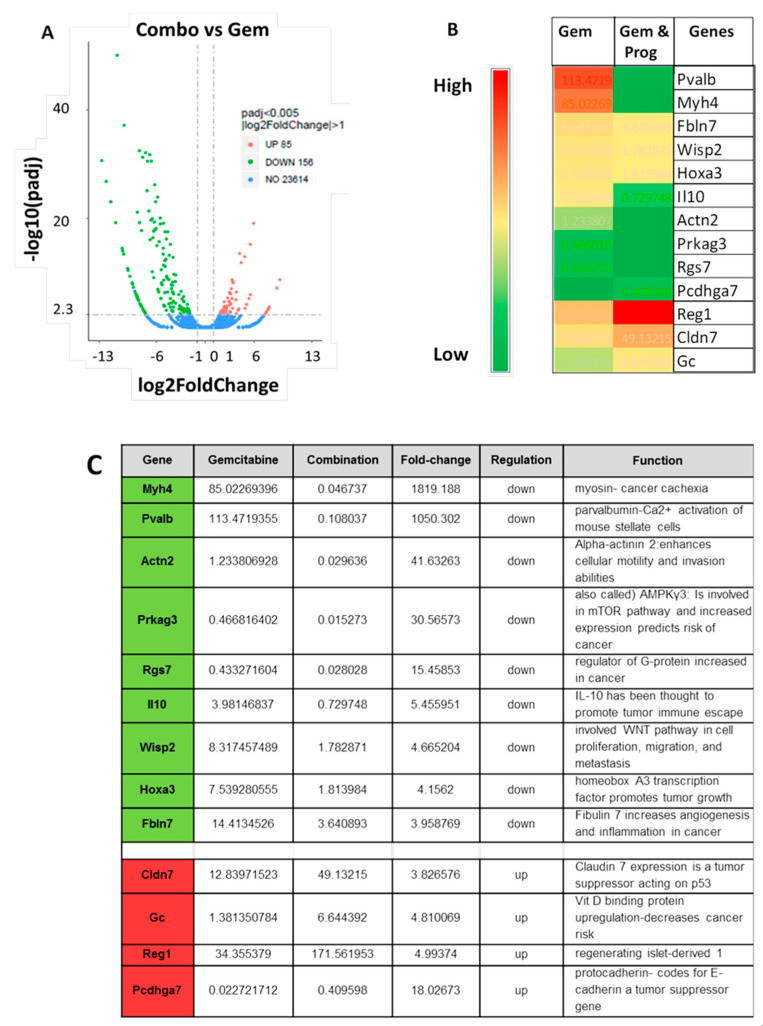
Differentially expressed genes (DEGs) in tumors of mice treated with gemcitabine therapy versus combination therapy. (**A**) Volcano plot of 85 genes that are upregulated Log2-fold in the combination therapy group compared to gemcitabine monotherapy are shown in red dots to the right of the midline. Those 156 genes that are downregulated by the addition of proglumide to gemcitabine in pancreatic tumors are shown in green dots to the left. (**B**) Heat map of 13 novel genes that differentially expressed in pancreas tumors of mice treated with the combination therapy compared to gemcitabine monotherapy. Scale bar is placed to the left with red and green representing highly versus lowly expressed genes based on FPKM values, respectively. (**C**) Table of the 13 novel selected differentially expressed genes that are significantly up or downregulated by combined therapy compared to gemcitabine monotherapy and their known function.

Differentially expressed genes (DEGs) were analyzed by RNAseq between the tumors of mice treated with gemcitabine monotherapy or tumors of those treated with the combination of gemcitabine and proglumide. A volcano plot of the 85 genes that are upregulated Log2-fold in the combination therapy group compared to gemcitabine monotherapy and the 156 genes that are downregulated with addition of proglumide are shown in Figure 5A. All of the data on the 241 DEGs examined in the two groups are shown in Supplementary Materials Table S3. Thirteen novel genes were identified that previously have not been reported to be altered by proglumide therapy and a heat map of these highlighted genes is shown (Figure 5B). A description of these 13 genes and their function is described in Figure 5C.

### 3.6. Combination Therapy Increases Gemcitabine Uptake into Tumors and Alters microRNA Blood Biomarker Profile

**Figure 6:**
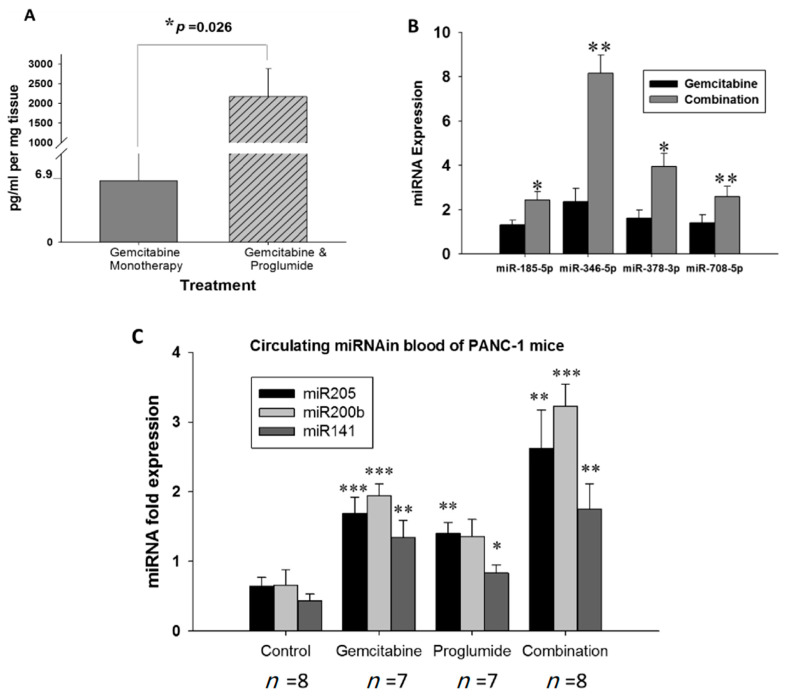
Combination therapy increases gemcitabine uptake into tumors and alters microRNA blood biomarker profile. (**A**) Measurement of gemcitabine by mass spectrometry in mT3-2D tumors. Tumors of mice treated with the combination of proglumide show increased uptake of measured gemcitabine (* *p* = 0.0264; two-tailed *t*-test). (**B**) Expression of miRNA markers associated with: fibrinolysis measured from circulating blood from mice bearing mT3-2D immunocompetent subcutaneous tumors; (* *p* < 0.05, ** *p* < 0.01). (**C**) Expression of miRNA markers associated with invasion measured from mice bearing PANC-1 orthotopic tumors; (* *p* < 0.05, ** *p* < 0.01; *** *p* < 0.005).

Gemcitabine concentration was measured in the larger mT3-2D tumors of mice treated with gemcitabine monotherapy or gemcitabine in combination with proglumide by Multiple Reaction Monitoring (MRM) based ultra-performance liquid chromatography (UPLC) coupled with Xevo-TQSmass spectrometer analytical assay. Mean gemcitabine levels (pg/mL per mg of tumor tissue) were significantly higher in the tumors of mice treated with the combination therapy (Figure 6A). These results indicate that proglumide therapy enhances the uptake of gemcitabine into PDAC tumors possibly by decreasing the fibrosis in the PDAC TME.

### 3.7. Measurement of Circulating microRNAs Confirm Decreased Fibrosis and Invasion upon Gemcitabine and Proglumide Combination Therapy

The microRNAs (miR) miR-185-5p, miR-346-5p, miR-378-3p, miR-708-5p are known to inhibit fibrosis from stellate cells [46,47,48,49]. These were significantly upregulated in the peripheral blood of mice treated with the combination therapy in the mT3-2D immunocompetent PDAC model (Figure 6B). miR-141, miR205, and miR-200b are members of the miR-200 family, which are known to be negative regulators of EMT to maintain an epithelial phenotype [50,51]. We found these three markers to be significantly upregulated in combination treated mice compared to the gemcitabine monotherapy mice in the PANC-1 immune deficient PDAC model (Figure 6C).

## 4. Discussion

The new finding from this investigation shows that monotherapy with a CCK receptor antagonist, proglumide, is as effective as gemcitabine for treatment of pancreatic cancer and the combination regimen may exhibit increased inhibitory effects. One reason for the effectiveness of proglumide monotherapy in pancreatic cancer is in part related to its receptor targeted-specific inhibition of the CCK receptor activation. When proglumide and gemcitabine are administered in combination to mice bearing pancreatic tumors, there is an inhibitory effect with decreased tumor growth, prolonged survival, and markedly decreased metastases compared to when either drug is administered as a single agent. Furthermore, tumors of mice treated with a combination regimen exhibited a significantly greater uptake of intratumoral gemcitabine by mass spectroscopy as compared to gemcitabine monotherapy. A possible explanation for this increased uptake of gemcitabine into the tumors of mice treated with the combination therapy is that proglumide treatment decreased intratumoral fibrosis. This dense desmoplastic reaction in the TME is thought to impede the uptake of chemotherapeutic agents and is thus partially responsible for increased drug resistance [52].

Pancreatic cancer is considered a “cold tumor”, lacking CD8^+^ TILs, and use of immune checkpoint antibodies has thus far largely failed in PDAC [53]. Prior studies have demonstrated arginase-positive cells contribute to immune suppression in cancer with arginase also being a well-known marker for M2-polarized macrophages. These immunosuppressive cells also facilitate angiogenesis and tumor cell mobility by remodeling the ECM [54,55,56]. Therapy with proglumide changed the immunologically “cold tumors” to “hot” or sensitive tumors by allowing for the influx of CD8^+^ T lymphocytes and decreasing arginase-positive cells. This change in the immune phenotype of the TME was a characteristic feature only attributed to proglumide and not to gemcitabine. The alteration in tumor immune cell signature from proglumide therapy improved the effectiveness of gemcitabine.

Gemcitabine resistance is a common problem amongst pancreatic cancer patients. Previous studies demonstrated that other gemcitabine metabolites, such as 2′,2′-difluorodeoxyuridine (dFdU) compete with gemcitabine (dFdC) for entry into malignant cells [57]. A recent study showed that TAMs could release other pyrimidine nucleosides to confer gemcitabine resistance in malignant cells [58]. PANC-1 human pancreatic cancer is often used as a model when studying gemcitabine resistance [59] and our results demonstrate that PANC-1 gemcitabine resistance can be overcome with a combination therapy with proglumide. Along with the decrease in arginase-positive cells in our immunocompetent model, these data further support the use of proglumide in combination with gemcitabine for potential clinical use.

PSCs have been identified as the main cellular source of the ECM protein deposition within the PDAC TME to stimulate tumor progression [6,26,60,61,62]. These cancer-associated fibroblasts (CAFs) are believed to be derived from mesenchymal cells of different origins that are either resident or recruited to the pancreas by neoplastic cells [63]. Two distinct populations of CAFs have been identified: (1) inflammatory fibroblasts and (2) myofibroblasts [63]. The elimination of SHH signaling eliminates the myofibroblasts, which predominately express alpha-smooth muscle actin (α-SMA), within the TME and rendered PDAC more aggressive and metastatic [64]. Contrarily, strategies to decrease inflammatory fibroblasts and FAP-positive fibroblasts are associated with decreased cancer growth and metastases [65,66]. Our results demonstrate that the decrease in intratumoral fibrosis by proglumide therapy is associated with a decrease in the expression of FAP. We previously showed that immune competent mice bearing syngeneic mT5-2D pancreatic tumors also exhibited decreased intratumoral fibrosis [21] when treated with proglumide, and analysis of these tumors did not demonstrate any decrease in immunoreactivity to α-SMA. This finding suggests that proglumide therapy preferentially decreases the expression of FAP and inflammatory CAFs. Matrix metalloproteases (MMPs) comprise several tightly regulated classes of proteases that play important roles in tumor progression and the metastatic process by facilitating ECM degradation [67,68,69]. We previously found that *Mmp* gene expression was altered in tumor-bearing mice by proglumide [21]. Our current results support previous findings that proglumide alters the fibrosis of the TME by decreasing FAP-expressing CAFs and rendering the TME more susceptible to chemotherapy.

Histology confirmed that proglumide reversed inflammation and fibrosis in this model. Differential expression of multiple miRNAs between the proglumide-treated and untreated PBS vehicle control were identified, suggesting that miRNAs might be also involved in regulation of a signaling pathway downstream from the CCK-B receptor. Strikingly, a significant portion of miRNAs from the panel have been reported for their implication in regulation of inflammation, fibrosis and oncogenesis. Among others, miR-185-5p, miR-346-5p, miR-378a-3p, miR-708-5p [46,47,48,49] are upregulated by proglumide and have been clearly shown to inhibit fibrosis. In the case of miR-185-5p, miR-378a-3p, miR-708-5p this effect is through inhibition of stellate cell activation. In the prior study, the changes in fibrosis associated miRNAs were analyzed in pancreatic tissue. The important finding in the current study of increased circulating levels in the peripheral blood of selective microRNAs associated with decreased fibrosis (miR-185-5p, miR-346-5p, miR-378a-3p) implies that these miRNAs may have potential use as biomarkers for non-invasively monitoring the effects of proglumide on fibrosis in pancreatic tumors.

An exciting new area of research involves the understanding and manipulation of the circulating miRNAs in order to prevent metastases [70]. Within the primary tumor, miRNAs have been shown to regulate EMT by phenotypic assays and via direct targets known to be involved in the EMT pathway. Recently, the five member miR-200 family (miR-141, -200a, -200b, -200c, and -429) and miR-205 have been identified as EMT-suppressive or ‘tumor-suppressive’ miRNAs directly targeting *ZEB1* and *ZEB2* [71,72]. The miR-200-ZEB1-E-cadherin axis has been clarified as a crucial pathway downstream of TGF-β in EMT while reciprocal repression between *ZEB1* and the miR-200 family has recently been reported to promote EMT and invasion in cancer cells [71,73,74]. This new class of tumor-suppressive miRNAs (TS-miRNAs) with in vitro or in vivo delivery technology along with the development of EMT inhibitors may provide novel strategies for the prevention and treatment of cancers. The transcription factors and proteins that regulate EMT were significantly altered when gemcitabine and proglumide were co-administered, and this result supports that this regimen decreases the potential of a primary tumor for metastasis. An important finding from our work is that the miRNAs (miR-200b, miR-205, and miR-141) that regulate these intratumoral transcription factors can be measured in the peripheral blood and correlated with the metastatic potential of a tumor. An innovative finding of our investigation is the potential to develop noninvasive miRNA biomarker panels to evaluate response to therapy with markers that correlate with decreased EMT and intratumoral fibrosis.

RNAseq results identified novel differentially expressed genes in tumors of mice receiving combination therapy compared to gemcitabine monotherapy. The myosin heavy chain (MyHC) expression is high in the tumors of gemcitabine monotherapy-treated mice and this gene is significantly decreased with proglumide. MyHC overexpression has been recently implicated in cancer cachexia [75]. The direct mechanism of how proglumide can potentially help to prevent cancer cachexia is unknown, but proglumide can interact with CCK receptors in the brain to prevent satiety and improve appetite. Another gene, parvalbumin, that is a calcium-binding protein involved in pancreatic stellate cells activation and proliferation, ref. [76] was significantly downregulated by proglumide therapy; perhaps this in part contributed to the decreased fibrosis observed in our models. Interleukin-10 (IL-10) is an immunosuppressive cytokine that is thought to promote tumor ‘immune escape’ by diminishing anti-tumor immune response in the TME [77]. Downregulation of IL-10 expression by proglumide may explain the mechanism involved with the change in the tumor immune cell signature we observed in the tumors. Xing et al. [78] have shown that downregulation of IL-10 augments gemcitabine chemosensitization in human pancreatic cancer cells. Four novel genes were up-regulated in tumors of mice treated with proglumide including claudin-7 (Cldn7), the vitamin D binding protein (Gc), regenerating islet-derived 1 (Reg1), and protocadherin (Pcdhag7). Both claudin-7 [79] and protocadherin [80] are tumor suppressor genes that act on p53 and E-cadherin, respectively. The third novel gene that was up-regulated by proglumide was GC, vitamin D binding protein. The Reg1 gene has been described in pancreatic cancer, where it has recently been shown to inhibit islet stellate cell activation [81]. An epidemiologic study showed that higher vitamin D binding protein was associated with a significantly reduced risk of pancreatic cancer (OR = 0.33, 95% CI 0.16–0.70) [82].

## 5. Conclusions

Our data demonstrate a novel combination therapeutic regimen for pancreatic cancer that decreases metastasis and tumor growth by decreasing fibrosis and altering the immune response. Since most subjects diagnosed with PDAC are not candidates for surgery and are treated with chemotherapy, our approach provides rationale for possibly combining standard of care chemotherapy with the CCK receptor antagonist, proglumide. Another beneficial outcome of the combined regimen is the decrease in metastasis, which is the cause of death in most patients with pancreatic cancer. Proglumide is an older orally bioavailable drug with a broad safety profile that was originally developed for peptic ulcer disease [83]; therefore, extending its therapeutic use to PDAC in combination with standard gemcitabine-based chemotherapy is feasible to improve treatment of this disease.

## Data Availability

The datasets used and analyzed during the current study are available.

## Supplementary Materials

The following are available online at https://www.mdpi.com/article/10.3390/cancers13194949/s1, Figure S1: Study Design, Table S1. qRT-PCR primers and miRNA primers, Supplementary Methods: including Mass Spectroscopy Methods, Reagents and chemicals, Preparation of tissue samples, Liquid Chromatography-Mass Spectrometry, Figure S2: UPLC-MRM MS analysis of Gemcitabine mT3-2D immunocompetent subcutaneous mouse tumors treated with Gemcitabine alone or in combination therapy with Proglumide, Table S2: Number of metastases in each in specific organs, Figure S3. Histologic confirmation of pancreatic cancer metastases, Table S3: Differentially expressed genes by RNAseq comparing gemcitabine (GEM) to combination therapy (Comb). Figure S4: Original western blots.

## References

[1] Ryan D.P., Hong T.S., Bardeesy N. (2014). Pancreatic adenocarcinoma. N. Engl. J. Med..

[2] Ansari D., Tingstedt B., Andersson B., Holmquist F., Sturesson C., Williamson C., Sasor A., Borg D., Bauden M., Andersson R. (2016). Pancreatic cancer: Yesterday, today and tomorrow. Future Oncol..

[3] Amrutkar M., Gladhaug I.P. (2017). Pancreatic Cancer Chemoresistance to Gemcitabine. Cancers.

[4] Patnaik A., Kang S.P., Rasco D., Papadopoulos K.P., Elassaiss-Schaap J., Beeram M., Drengler R., Chen C., Smith L., Espino G. (2015). Phase I Study of Pembrolizumab (MK-3475; Anti-PD-1 Monoclonal Antibody) in Patients with Advanced Solid Tumors. Clin. Cancer Res..

[5] Zeng S., Pottler M., Lan B., Grutzmann R., Pilarsky C., Yang H. (2019). Chemoresistance in Pancreatic Cancer. Int. J. Mol. Sci..

[6] Apte M.V., Wilson J.S., Lugea A., Pandol S.J. (2013). A starring role for stellate cells in the pancreatic cancer microenvironment. Gastroenterology.

[7] Fang H., Declerck Y.A. (2013). Targeting the tumor microenvironment: From understanding pathways to effective clinical trials. Cancer Res..

[8] Von Hoff D.D., Ramanathan R.K., Borad M.J., Laheru D.A., Smith L.S., Wood T.E., Korn R.L., Desai N., Trieu V., Iglesias J.L. (2011). Gemcitabine plus nab-paclitaxel is an active regimen in patients with advanced pancreatic cancer: A phase I/II trial. J. Clin. Oncol..

[9] Conroy T., Desseigne F., Ychou M., Bouche O., Guimbaud R., Becouarn Y., Adenis A., Raoul J.L., Gourgou-Bourgade S., de la Fouchardiere C. (2011). FOLFIRINOX versus gemcitabine for metastatic pancreatic cancer. N. Engl. J. Med..

[10] Hingorani S.R., Harris W.P., Beck J.T., Berdov B.A., Wagner S.A., Pshevlotsky E.M., Tjulandin S.A., Gladkov O.A., Holcombe R.F., Korn R. (2016). Phase Ib Study of PEGylated Recombinant Human Hyaluronidase and Gemcitabine in Patients with Advanced Pancreatic Cancer. Clin. Cancer Res..

[11] Van C.E., Tempero M.A., Sigal D., Oh D.Y., Fazio N., Macarulla T., Hirte E., Hammel P., Hendifar A.E., Bates S.E. (2020). Randomized Phase III Trial of Pegvorhyaluronidase Alfa with Nab-Paclitaxel Plus Gemcitabine for Patients with Hyaluronan-High Metastatic Pancreatic Adenocarcinoma. J. Clin. Oncol..

[12] Noel M., O’Reilly E.M., Wolpin B.M., Ryan D.P., Bullock A.J., Britten C.D., Linehan D.C., Belt B.A., Gamelin E.C., Ganguly B. (2020). Phase 1b study of a small molecule antagonist of human chemokine (C-C motif) receptor 2 (PF-04136309) in combination with nab-paclitaxel/gemcitabine in first-line treatment of metastatic pancreatic ductal adenocarcinoma. Investig. New Drugs.

[13] Hecht J.R., Papadopoulos K.P., Falchook G.S., Patel M.R., Infante J.R., Aljumaily R., Wong D.J., Autio K.A., Wainberg Z.A., Bauer T.M. (2021). Immunologic and tumor responses of pegilodecakin with 5-FU/LV and oxaliplatin (FOLFOX) in pancreatic ductal adenocarcinoma (PDAC). Investig. New Drugs.

[14] Nazemi M., Rainero E. (2020). Cross-Talk between the Tumor Microenvironment, Extracellular Matrix, and Cell Metabolism in Cancer. Front. Oncol..

[15] Martinez-Bosch N., Vinaixa J., Navarro P. (2018). Immune Evasion in Pancreatic Cancer: From Mechanisms to Therapy. Cancers.

[16] Dufresne M., Seva C., Fourmy D. (2006). Cholecystokinin and gastrin receptors. Physiol. Rev..

[17] Berna M.J., Jensen R.T. (2007). Role of CCK/gastrin receptors in gastrointestinal/metabolic diseases and results of human studies using gastrin/CCK receptor agonists/antagonists in these diseases. Curr. Top. Med. Chem..

[18] Hahne W.F., Jensen R.T., Lemp G.F., Gardner J.D. (1981). Proglumide and benzotript: Members of a different class of cholecystokinin receptor antagonists. Proc. Natl. Acad. Sci. USA.

[19] Smith J.P., Cooper T.K., McGovern C.O., Gilius E.L., Zhong Q., Liao J., Molinolo A.A., Gutknd J.S., Matter G.L. (2014). Cholecystokinin receptor antagonist halts progression of pancreatic cancer precursor lesions and fibrosis in mice. Pancreas.

[20] Smith J.P., Liu G., Soundararajan V., Zagon I.S. (1994). Identification and characterization of CCK-B/gastrin receptors in human pancreatic cancer cell lines. Am. J. Physiol..

[21] Nadella S., Burks J., Al-Sabban A., Inyang G., Wang J., Tucker R.D., Zamanis M.E., Bukowski W., Shivapurkar N., Smith J.P. (2018). Dietary fat stimulates pancreatic cancer growth and promotes fibrosis of the tumor microenvironment through the cholecystokinin receptor. Am. J. Physiol. Gastrointest. Liver Physiol..

[22] Smith J.P., Solomon T.E. (2014). Cholecystokinin and pancreatic cancer: The chicken or the egg?. Am. J. Physiol. Gastrointest. Liver Physiol..

[23] Singh P., Owlia A., Espeijo R., Dai B. (1995). Novel gastrin receptors mediate mitogenic effects of gastrin and processing intermediates of gastrin on Swiss 3T3 fibroblasts. Absence of detectable cholecystokinin (CCK)-A and CCK-B receptors. J. Biol. Chem..

[24] Berna M.J., Seiz O., Nast J.F., Benten D., Blaker M., Koch J., Lohse A.W., Pace A. (2010). CCK1 and CCK2 receptors are expressed on pancreatic stellate cells and induce collagen production. J. Biol. Chem..

[25] Phillips P.A., Yang L., Shulkes A., Vonlaufen A., Poljak A., Bustamante S., Warren A., Xu Z., Guilhaus M., Pirola R. (2010). Pancreatic stellate cells produce acetylcholine and may play a role in pancreatic exocrine secretion. Proc. Natl. Acad. Sci. USA.

[26] Apte M.V., Park S., Phillips P.A., Santucci N., Goldstein D., Kumar R.K., Ramm G.A., Buchler M., Friess H., McCarroll J.A. (2004). Desmoplastic reaction in pancreatic cancer: Role of pancreatic stellate cells. Pancreas.

[27] Boj S.F., Hwang C.I., Baker L.A., Chio I.I.C., Engle D.D., Corbo V., Jager M., Ponz-Sarvise M., Titiac H., Spector M.S. (2015). Organoid models of human and mouse ductal pancreatic cancer. Cell.

[28] LaConti J.J., Shivapurkar N., Preet A., Mays A.D., Peran I., Kim S.E., Marshall J.L., Riegel A.T., Wellstein A. (2011). Tissue and serum microRNAs in the Kras(G12D) transgenic animal model and in patients with pancreatic cancer. PLoS ONE.

[29] Shivapurkar N., Weiner L.M., Marshall J.L., Madhavan S., Mays A.D., Juhl H., Wellstein A. (2014). Recurrence of early stage colon cancer predicted by expression pattern of circulating microRNAs. PLoS ONE.

[30] Shivapurkar N., Vietsch E.E., Carney E., Isaacs C., Wellstein A. (2017). Circulating microRNAs in patients with hormone receptor-positive, metastatic breast cancer treated with dovitinib. Clin. Transl. Med..

[31] Osborne N., Sundseth R., Gay M.D., Cao H., Tucker R.D., Nadella S., Wang S., Liu X., Kroemer A., Sutton L. (2019). Vaccine Against Gastrin, Polyclonal Antibody Stimulator, Decreases Pancreatic Cancer Metastases. Am. J. Physiol. Gastrointest. Liver Physiol..

[32] Balachandran V.P., Luksza M., Zhao J.N., Makarov V., Moral J.A., Remark R., Herbst B., Askan G., Bhanot U., Senbabaoglu Y. (2017). Identification of unique neoantigen qualities in long-term survivors of pancreatic cancer. Nature.

[33] Rodriguez-Aznar E., Wiesmuller L., Sainz B., Hermann P.C. (2019). EMT and Stemness-Key Players in Pancreatic Cancer Stem Cells. Cancers.

[34] Wang S., Huang S., Sun Y.L. (2017). Epithelial-Mesenchymal Transition in Pancreatic Cancer: A Review. BioMed Res. Int..

[35] Satelli A., Li S. (2011). Vimentin in cancer and its potential as a molecular target for cancer therapy. Cell Mol. Life Sci..

[36] Handra-Luca A., Hong S.M., Walter K., Wolfgang C., Hruban R., Goggins M. (2011). Tumour epithelial vimentin expression and outcome of pancreatic ductal adenocarcinomas. Br. J. Cancer.

[37] Zhang P., Sun Y., Ma L. (2015). ZEB1: At the crossroads of epithelial-mesenchymal transition, metastasis and therapy resistance. Cell Cycle.

[38] Wang Y., Shi J., Chai K., Ying X., Zhou B.P. (2013). The Role of Snail in EMT and Tumorigenesis. Curr. Cancer Drug Targets.

[39] Hotz B., Arndt M., Dullat S., Bhargava S., Buhr H.J., Hotz H.G. (2007). Epithelial to mesenchymal transition: Expression of the regulators snail, slug, and twist in pancreatic cancer. Clin. Cancer Res..

[40] Mylavarapu S., Kumar H., Kumari S., Sravanthi L.S., Jain M., Basu A., Biswas M., Mylavarapu S.V.S., Das A., Roy M. (2019). Activation of Epithelial-Mesenchymal Transition and Altered beta-Catenin Signaling in a Novel Indian Colorectal Carcinoma Cell Line. Front. Oncol..

[41] Bierie B., Moses H.L. (2010). Transforming growth factor beta (TGF-beta) and inflammation in cancer. Cytokine Growth Factor Rev..

[42] Corbet C., Bastien E., Santiago de Jesus J.P., Dierge R., Martherus R., Linden C.V., Doix B., Degavre C., Guilbaud C., Petit L. (2020). TGFbeta2-induced formation of lipid droplets supports acidosis-driven EMT and the metastatic spreading of cancer cells. Nat. Commun..

[43] Mu G., Ding Q., Li H., Zhang L., Zhang L., He K., Wu L., Deng Y., Yang D., Wu L. (2018). Gastrin stimulates pancreatic cancer cell directional migration by activating the Galpha12/13-RhoA-ROCK signaling pathway. Exp. Mol. Med..

[44] Pedone E., Marucci L. (2019). Role of beta-Catenin Activation Levels and Fluctuations in Controlling Cell Fate. Genes.

[45] Zhuang K., Yan Y., Zhang X., Zhang J., Zhang L., Han K. (2016). Gastrin promotes the metastasis of gastric carcinoma through the beta-catenin/TCF-4 pathway. Oncol. Rep..

[46] Zhang Y., Xiao H.Q., Wang Y., Yang Z.S., Dai L.J., Xu Y.C. (2015). Differential expression and therapeutic efficacy of microRNA-346 in diabetic nephropathy mice. Exp. Ther. Med..

[47] Hyun J., Wang S., Kim J., Rao K.M., Park S.Y., Chung I., Ha C.S., Kim S.W., Yum Y.H., Jung Y. (2016). MicroRNA-378 limits activation of hepatic stellate cells and liver fibrosis by suppressing Gli3 expression. Nat. Commun..

[48] Zhou L., Liu S., Han M., Ma Y., Feng S., Zhao J., Lu H., Yuan X., Cheng J. (2018). miR-185 Inhibits Fibrogenic Activation of Hepatic Stellate Cells and Prevents Liver Fibrosis. Mol. Ther. Nucleic Acids..

[49] Yang J., Tao Q., Zhou Y., Chen Q., Li L., Hu S., Liu Y., Zhang Y., Shu J., Zhang X. (2020). MicroRNA-708 represses hepatic stellate cells activation and proliferation by targeting ZEB1 through Wnt/beta-catenin pathway. Eur. J. Pharmacol..

[50] Abba M.L., Patil N., Leupold J.H., Allgayer H. (2016). MicroRNA Regulation of Epithelial to Mesenchymal Transition. J. Clin. Med..

[51] Yu K.R., Lee S., Jung J.W., Hong I.S., Kim H.S., Seo Y., Shin T.H., Kang K.S. (2013). MicroRNA-141-3p plays a role in human mesenchymal stem cell aging by directly targeting ZMPSTE24. J. Cell Sci..

[52] Erkan M., Michalski C.W., Rieder S., Reiser-Erkan C., Abiatari I., Kolb A., Giese N.A., Esposito I., Friess H., Kleeff J. (2008). The activated stroma index is a novel and independent prognostic marker in pancreatic ductal adenocarcinoma. Clin. Gastroenterol. Hepatol..

[53] Brahmer J.R., Tykodi S.S., Chow L.Q., Hwu W.J., Topalian S.L., Hwu P., Drake C.G., Camacho L.H., Kauh J., Odunsi K. (2012). Safety and activity of anti-PD-L1 antibody in patients with advanced cancer. N. Engl. J. Med..

[54] Vonderheide R.H., Bayne L.J. (2013). Inflammatory networks and immune surveillance of pancreatic carcinoma. Curr. Opin. Immunol..

[55] Zheng L., Xue J., Jaffee E.M., Habtezion A. (2013). Role of immune cells and immune-based therapies in pancreatitis and pancreatic ductal adenocarcinoma. Gastroenterology.

[56] Condeelis J., Pollard J.W. (2006). Macrophages: Obligate partners for tumor cell migration, invasion, and metastasis. Cell.

[57] Derissen E.J.B., Huitema A.D.R., Rosing H., Schellens J.H.M., Beijnen J.H. (2018). Intracellular pharmacokinetics of gemcitabine, its deaminated metabolite 2′,2′-difluorodeoxyuridine and their nucleotides. Br. J. Clin. Pharmacol..

[58] Halbrook C.J., Pontious C., Kovalenko I., Lapenyte L., Dreyer S., Lee H.J., Thurston G., Zhang Y., Lazarus J., Sajjakulnukit P. (2019). Macrophage-Released Pyrimidines Inhibit Gemcitabine Therapy in Pancreatic Cancer. Cell Metab..

[59] Rejiba S., Reddy L.H., Bigand C., Parmentier C., Couvreur P., Hajri A. (2011). Squalenoyl gemcitabine nanomedicine overcomes the low efficacy of gemcitabine therapy in pancreatic cancer. Nanomedicine.

[60] Farrow B., Albo D., Berger D.H. (2008). The role of the tumor microenvironment in the progression of pancreatic cancer. J. Surg. Res..

[61] Korc M. (2007). Pancreatic cancer-associated stroma production. Am. J. Surg..

[62] Moir J.A., Mann J., White S.A. (2015). The role of pancreatic stellate cells in pancreatic cancer. Surg. Oncol..

[63] Öhlund D., Handly-Santana A., Biffi G., Elyada E., Almeida A., Ponz-Sarvise M., Corbo V., Oni T.E., Hearn S.A., Lee E.J. (2017). Distinct populations of inflammatory fibroblasts and myofibroblasts in pancreatic cancer. J. Exp. Med..

[64] Rhim A.D., Oberstein P.E., Thomas D.H., Mirek E.T., Palermo C.F., Sastra S.A., Dekleva E.N., Saunders T., Becerra C.P., Tattersall I.W. (2014). Stromal elements act to restrain, rather than support, pancreatic ductal adenocarcinoma. Cancer Cell.

[65] Fan M.H., Zhu Q., Li H.H., Ra H.J., Majumdar S., Gulick D.L., Jerome J.A., Madsen D.H., Christofidou-Solomidou M., Speicher D.W. (2016). Fibroblast Activation Protein (FAP) Accelerates Collagen Degradation and Clearance from Lungs in Mice. J. Biol. Chem..

[66] Kraman M., Bambrough P.J., Arnold J.N., Roberts E.W., Magiera L., Jones J.O., Gopinathan A., Tuveson D.A., Fearon D.T. (2010). Suppression of antitumor immunity by stromal cells expressing fibroblast activation protein-alpha. Science.

[67] Egeblad M., Werb Z. (2002). New functions for the matrix metalloproteinases in cancer progression. Nat. Rev. Cancer.

[68] Gialeli C., Theocharis A.D., Karamanos N.K. (2011). Roles of matrix metalloproteinases in cancer progression and their pharmacological targeting. FEBS J..

[69] Kapoor C., Vaidya S., Wadhwan V., Hitesh, Kaur G., Pathak A. (2016). Seesaw of matrix metalloproteinases (MMPs). J. Cancer Res. Ther..

[70] Wu M., Wang G., Hu W., Yao Y., Yu X.F. (2019). Emerging roles and therapeutic value of exosomes in cancer metastasis. Mol. Cancer.

[71] Gregory P.A., Bert A.G., Paterson E.L., Barry S.C., Tsykn A., Farshid G., Vadas M.A., Khew-Goodall Y., Goodall G.J. (2008). The miR-200 family and miR-205 regulate epithelial to mesenchymal transition by targeting ZEB1 and SIP1. Nat. Cell Biol..

[72] Rawat M., Nighot M., Al-Sadi R., Gupta Y., Viszwapriya D., Yochun G., Koltun W., Ma T.Y. (2020). IL1B Increases Intestinal Tight Junction Permeability by Up-regulation of MIR200C-3p, Which Degrades Occludin mRNA. Gastroenterology.

[73] Bracken C.P., Gregory P.A., Kolesnikoff N., Bert A.G., Wang J., Shannon M.F., Goodall G.J. (2008). A double-negative feedback loop between ZEB1-SIP1 and the microRNA-200 family regulates epithelial-mesenchymal transition. Cancer Res..

[74] Park S.M., Gaur A.B., Lengyel E., Peter M.E. (2008). The miR-200 family determines the epithelial phenotype of cancer cells by targeting the E-cadherin repressors ZEB1 and ZEB2. Genes Dev..

[75] Yamada T., Ashida Y., Tatebayashi D., Abe M., Himori K. (2020). Cancer Cachexia Induces Preferential Skeletal Muscle Myosin Loss When Combined with Denervation. Front. Physiol..

[76] Won J.H., Zhang Y., Ji B., Logsdon C.D., Yule D.I. (2011). Phenotypic changes in mouse pancreatic stellate cell Ca^2+^ signaling events following activation in culture and in a disease model of pancreatitis. Mol. Biol. Cell.

[77] Mannino M.H., Zhu Z., Xiao H., Bai Q., Wakefield M.R., Fang Y. (2015). The paradoxical role of IL-10 in immunity and cancer. Cancer Lett..

[78] Xing H.B., Tong M.T., Wang J., Hu H., Zhai C.Y., Huang C.X., Li D. (2018). Suppression of IL-6 Gene by shRNA Augments Gemcitabine Chemosensitization in Pancreatic Adenocarcinoma Cells. BioMed Res. Int..

[79] Hou Y., Hou L., Liang Y., Zhang Q., Hong X., Wang Y., Huang X., Zhong T., Pang W., Xu C. (2020). The p53-inducible CLDN7 regulates colorectal tumorigenesis and has prognostic significance. Neoplasia.

[80] Liu Y., Peng K., Xie R., Zheng J., Guo J., Wei R., Yang H., Cai C., Wei Q. (2019). Protocadherin gamma-A7 is down-regulated in colorectal cancer and associated with the prognosis in patients with wild-type KRAS. Hum. Pathol..

[81] Xu W., Li W., Wang Y., Zha M., Yao H., Jones P.M., Sun Z. (2015). Regenerating islet-derived protein 1 inhibits the activation of islet stellate cells isolated from diabetic mice. Oncotarget.

[82] Weinstein S.J., Stolzenberg-Solomon R.Z., Kopp W., Rager H., Virtamo J., Albanes D. (2012). Impact of circulating vitamin D binding protein levels on the association between 25-hydroxyvitamin D and pancreatic cancer risk: A nested case-control study. Cancer Res..

[83] Miederer S.E., Lindstaedt H., Kutz K., Wuttke H. (1979). Efficient treatment of gastric ulcer with proglumide (Milid) in outpatients (double blind trial). Acta Hepatogastroenterol..

