# Supplementary material for: Cholecystokinin Receptor Antagonist Improves Efficacy of Chemotherapy in Murine Models of Pancreatic Cancer by Altering the Tumor Microenvironment
Source: Cancers (Basel). 2021 Sep 30;13(19):4949. doi: 10.3390/cancers13194949 (PMC8508339; doi:10.3390/cancers13194949)
Supplement: Supplementary file 1 [file cancers-13-04949-s001.zip › cancers-1379796 - supplementary maretials.pdf]

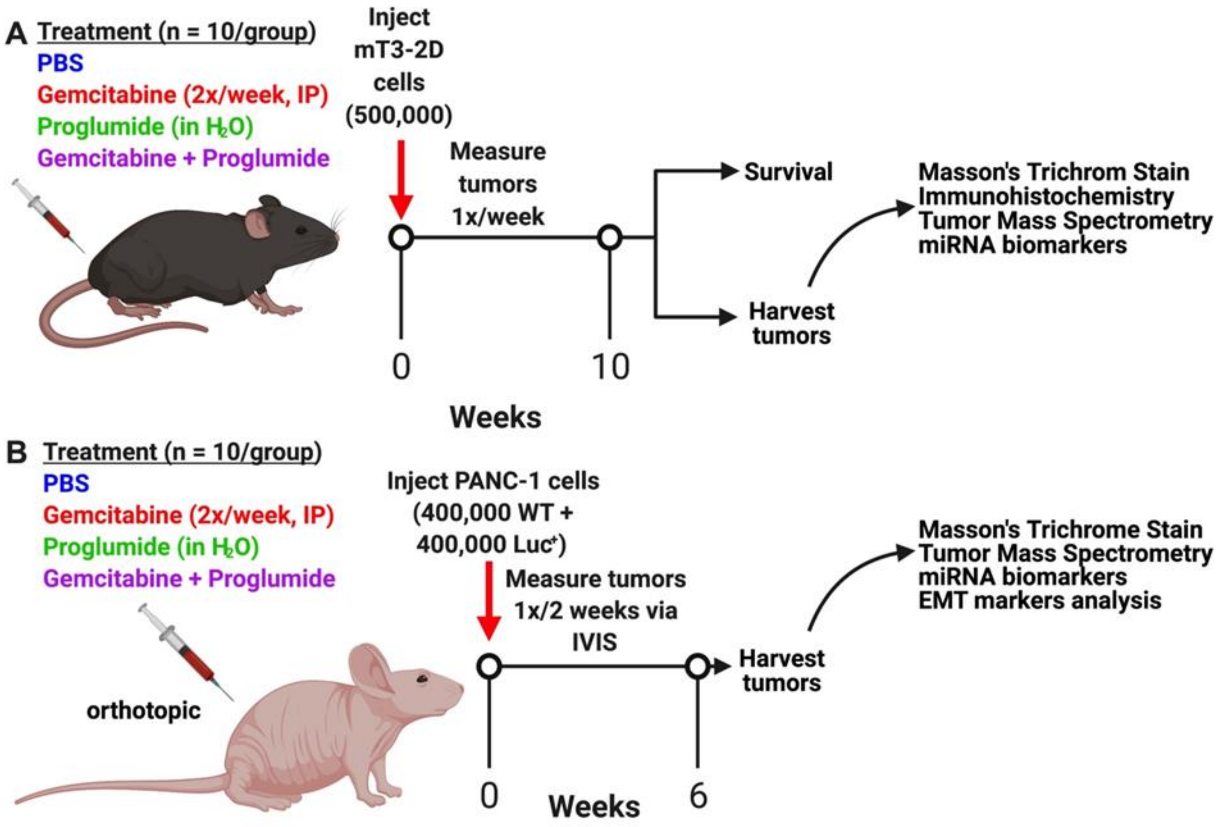

Figure S1. Study Design. (**A**) The first animal model used was an immunocompetent C57BL/6 with female mice injected subcutaneously with 500,000 mT3-2D syngeneic murine pancreatic cancer cells. This study was a survival study and tumors were euthanized when a diameter of 20mm was reached. Tumors were measured with calipers and the volume calculated by the formula: Lx W^2 x0.5. (**B**) The second animal model included immune deficient athymic nude male mice with orthotopic human PANC-1 tumors (800,000). Tumors were measured by luciferase flux in an IVIS machine. All the tumors in this study were harvested the same day after 6-weeks of therapy and metastases counted and dissected. In both studies there were 4 groups of mice (N=10 each) treated with PBS-control (0.1ml IP, 2X/week); gemcitabine monotherapy (100mg/kg, 100µl IP twice weekly); proglumide in drinking water (0.1 mg/ml), or the combination of gemcitabine and proglumide (same dose as monotherapy).

Table S1. qRT-PCR primers and miRNA primers.

**qRT-PCR primers**		
**Gene**	**Forward (5' → 3')**	**Reverse (5' → 3')**
Vimentin	TCCACACGCACCTACAGTCT	CCGAGGACCGGGTCACATA
Zeb1	GCTGGCAAGACAACGTGAAAG	GCCTCAGGATAAATGACGGC
Zeb2	GGGACAGATCAGCACCAAAT	GACCCAGAATGAGACAAGCG
SNAIL	GGAAGCCTAACTACAGCGAGCT	TCCCAGATGAGCATTGGCA
B-CATENIN	AACTTGCCACACGRGCAATC	GCTGGCTCAAGTCAAAGTCC
TGFBR2	GCTCCCAGCCTTCATCCTTT	CTTGAGCAATCAGGAGCCCA
Myh4	AGAGCCAAGAGGAAACTGGAGG	CTCGTCCTCAATCTTGCTCTGC
IL10	CGGGAAGACAATAACTGCACCC	CGGTTAGCAGTATGTTGTCCAGC
Pcdhga7	CAGCCAAGTTAGCTGTGAGAAAAG	GGCTTGAGAGAAACGCCAGTCA
GAPDH (H)	GTCTCCTCTGACTTCAACAGCG	ACCACCCTCTTGCTGTAGCCAA
Gapdh (m)	GTGAAGGTCGGAGTCAACGGATT	AGTGATGGCATGGACTGTGGTC

**miRNA**	miRNA sequence
miR-185-5p	5'-UGGAGAGAAAGGCAGUUCCUGA-3'
miR-346-5p	5'-UGUCUGCCCGAGUGCCUGCCUCU-3
miR-378-3p	5'-ACUGGACUUGGAGUCAGAAGGC-3'
miR-708-5p	5'-AAGGAGCUUACAAUCUAGCUGGG-3'
miR-141-5p	5′-CAUCUUCCAGUACAGUGUUGGA-3′
miR-200b-5p	5′-CAUCUUACUGGGCAGCAUUGGA-3′
miR-205-5p	5′-UCCUUCAUUCCACCGGAGUCUG-3′

Supplementary Methods

1. Mass Spectroscopy Methods:

1.1. Reagents and chemicals: The following reagents were purchased: solvents such as acetonitrile, isopropanol and methanol Optima grade (Fisher Scientific); high purity formic acid (99%) (Thermo-Scientific); gemcitabine human GMP grade (Sagent Pharmaceutical); and debrisoquine (Sigma- Aldrich).

1.2. Preparation of tissue samples: The tissue samples were stored at -80°C and thawed on ice (4°C). Tissue samples, (20 mg wet weight) were processed by the addition of extraction buffer (500 µL, methanol) containing internal standard (400 ng/ml, debrisoquine). The samples were vortexed for 30 seconds followed by homogenization for 1 minute. The samples were incubated on ice for 20 min followed by incubation at -20°C for 20 min. Sample were centrifuged at 13,000 rpm for 20 min at 4°C. 12.5 µL of the supernatant was taken and diluted up to 1 ml, vortexed well before transferring to the Mass Spec sample vial for LC-MS analysis. A pooled QC was generated by mixing 30 µL of each sample.

1.3. Liquid Chromatography-Mass Spectrometry: The LC gradient method started with 100% of mobile phase A (0.2% formic acid in water) that involved a gradient change from 0% B (0.2% formic acid in acetonitrile) to 80% phase B in 1.5 minutes after an initial lag phase of 1.5 minutes with flow rate changing from 0.2 ml/min to 0.35 ml/min at 1.5 min and to 0.5 ml/ml at 3 min. The column was maintained at 40°C and injection volume was kept at 5 µL. The column eluent was introduced directly into the TQS mass spectrometer by electrospray operating in positive mode at a capillary voltage of 3.00 kV and a sampling cone voltage of 70 V. The desolvation gas flow was set to 500 L/hour and the desolvation temperature was set to 500°C. The cone gas flow was 150 L/hour and the source temperature was set to 150 ^{o}C. The sample cone voltage and collision energies were optimized for the analyte to obtain maximum ion intensity for parent and daughter ions using “IntelliStart” feature of MassLynx software (Waters

Corporation). The instrument parameters were optimized to gain maximum specificity and sensitivity of ionization for the parent and daughter ions. Signal intensities from the MRM Q1>Q3 ion pairs for the drug gemcitabine (264.2>112) and DBQ (176.3>134.1, IS) were ranked to ensure selection of the most intense precursor and fragment ion pair for MRM-based quantitation. This approach resulted in selection of cone voltages and collision energies that maximized the generation of each fragment ion species.

A

B

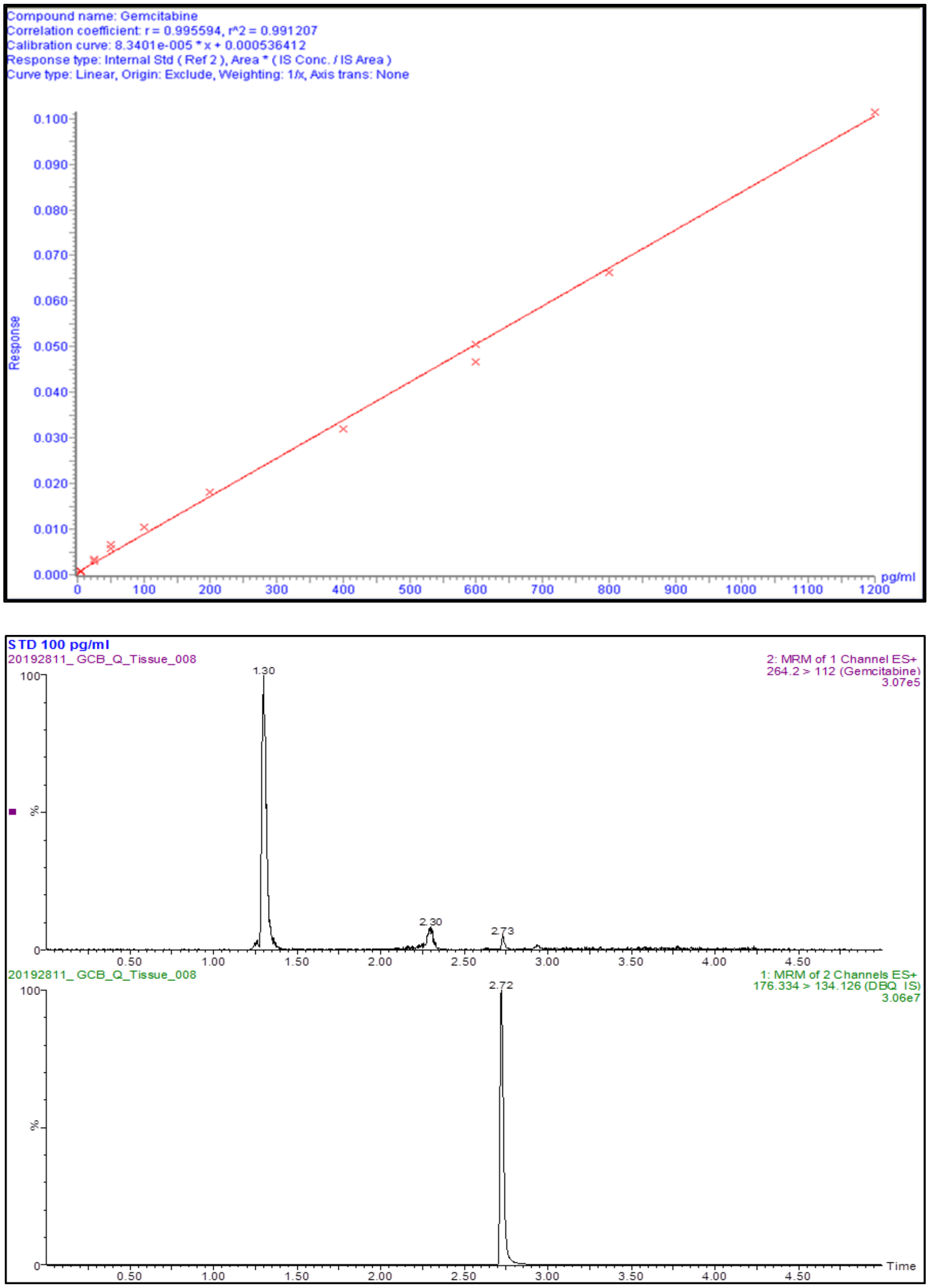

Figure S2. UPLC-MRM MS analysis of Gemcitabine mT3-2D immunocompetent subcutaneous mouse tumors treated with Gemcitabine alone or in combination therapy with Proglumide. (**A**) A standard calibration curve for gemcitabine concentrations is shown in the mentioned range. (**B**) A sample chromatogram of eluted gemcitabine and internal standard (DBQ) using the LC gradient is shown, as described in methods.

Table S2. Number of metastases in each in specific organs.

Group	Liver	Mesentery/ Peritoneum	Nodes	Spleen	Diaphragm	Abdominal Wall	Stomach	Colon
PBS	4	22	2	5	1	2	6	3
Gemcitabine	4	12	1	5	0	1	2	5
Proglumide	2	18	2	1	0	2	2	3
Combination	0	3	0	1	1	0	0	1

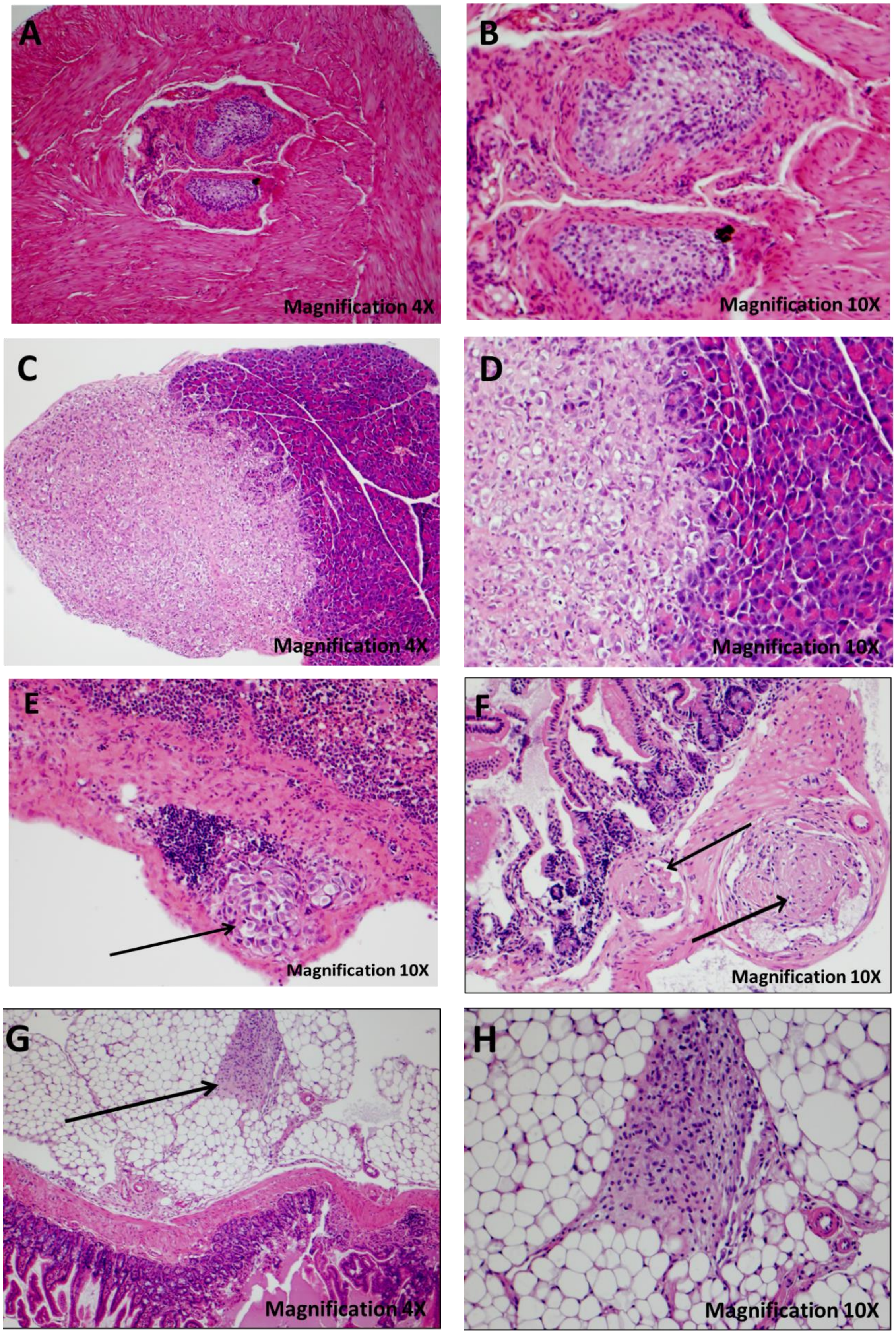
A
Magnification 4X
B
Magnification 10X
C
Magnification 4X
D
Magnification 10X
E
Magnification 10X
F
Magnification 10X
G
Magnification 4X
H
Magnification 10X

Figure S3. Histologic confirmation of pancreatic cancer metastases. (**A**) Tumor emboli are found in the abdominal wall between muscle fiber of a PBS-treated control mouse at low magnification (4X magnification). (**B**) The same image from panel (A) is magnified to demonstrate tow foci of metastases in the abdominal wall. (**C**) PANC-1 pancreatic cancer metastases to the liver from a mouse treated with proglumide monotherapy at low magnification. (**D**) Higher magnification of the liver metastases from panel (C) is shown with invading cancer cells (left) and normal liver parenchyma (right). (**E**) Metastasis to the splenic capsule is identified in a mouse treated with gemcitabine monotherapy (10X magnification). Arrow is pointing to the PANC-1 cancer cells. (**F**) Tumor metastases are shown in the mesentery adjacent to the colon. Arrows point to two areas of tumor cells. (**G**) Cancer metastases within the fatty peritoneum intestine is shown at a low magnification adjacent to the. (Arrow points to cancer cells). (**H**) Higher power magnification showing the PANC-1 cancer cells metastases in the peritoneum.

Original western blots Figures S4

FAP:

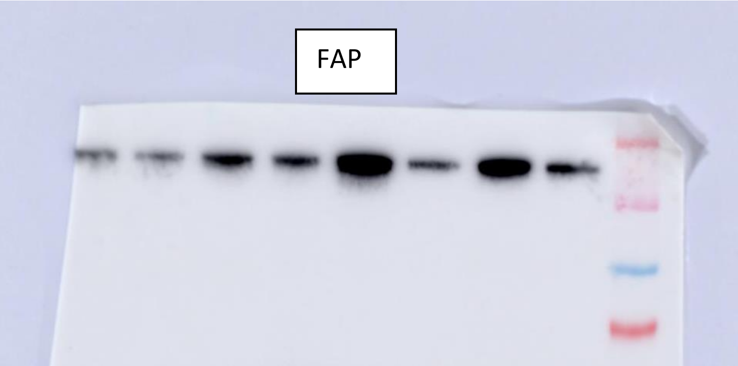

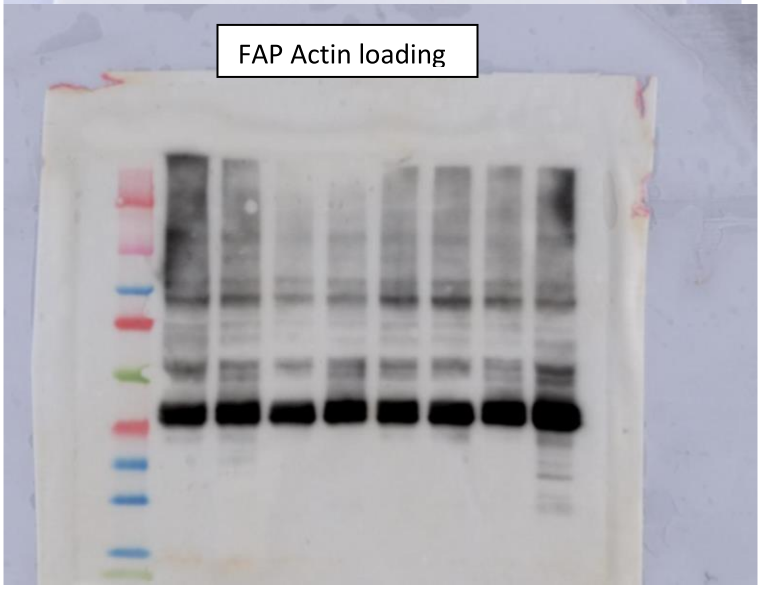

Phospho-paxillin

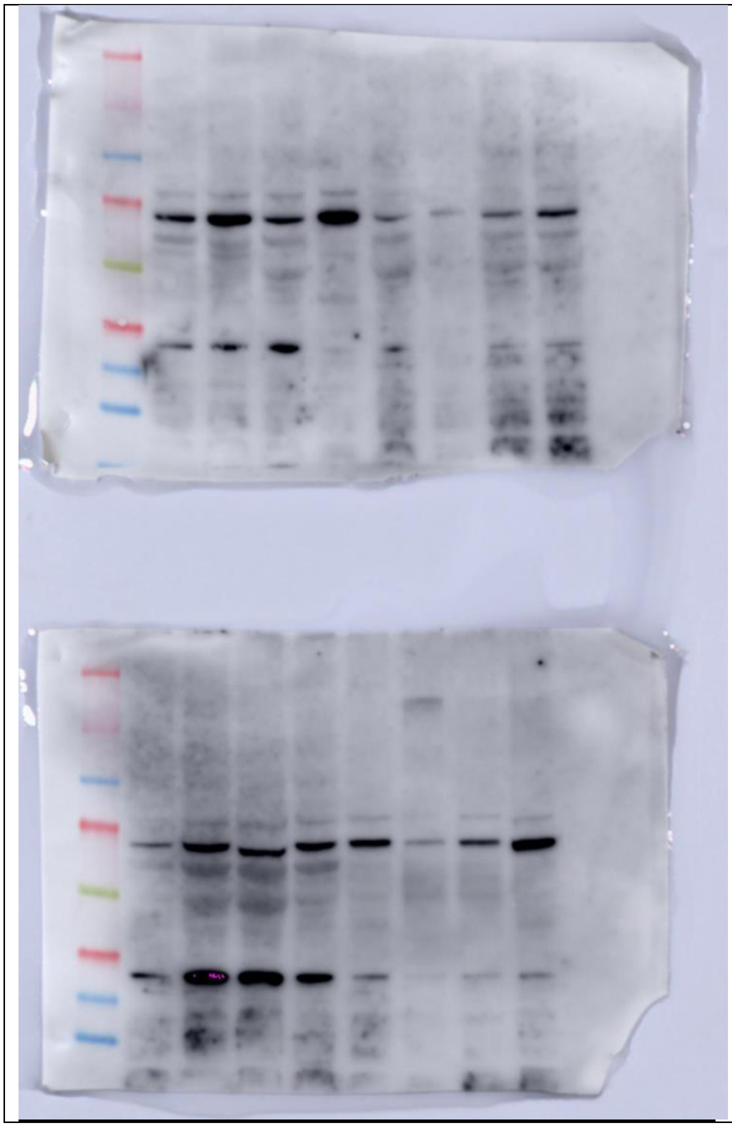

Actin loading Paxillin

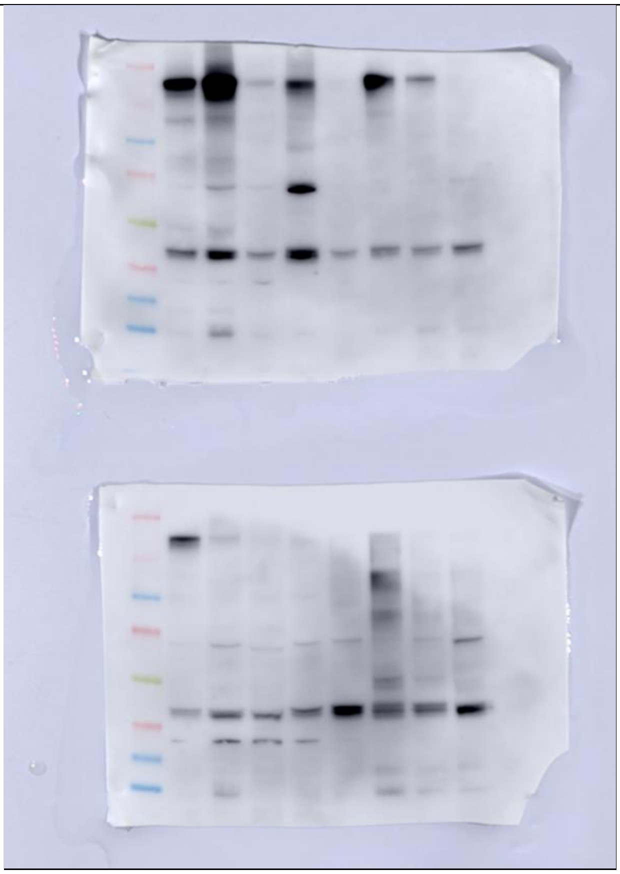

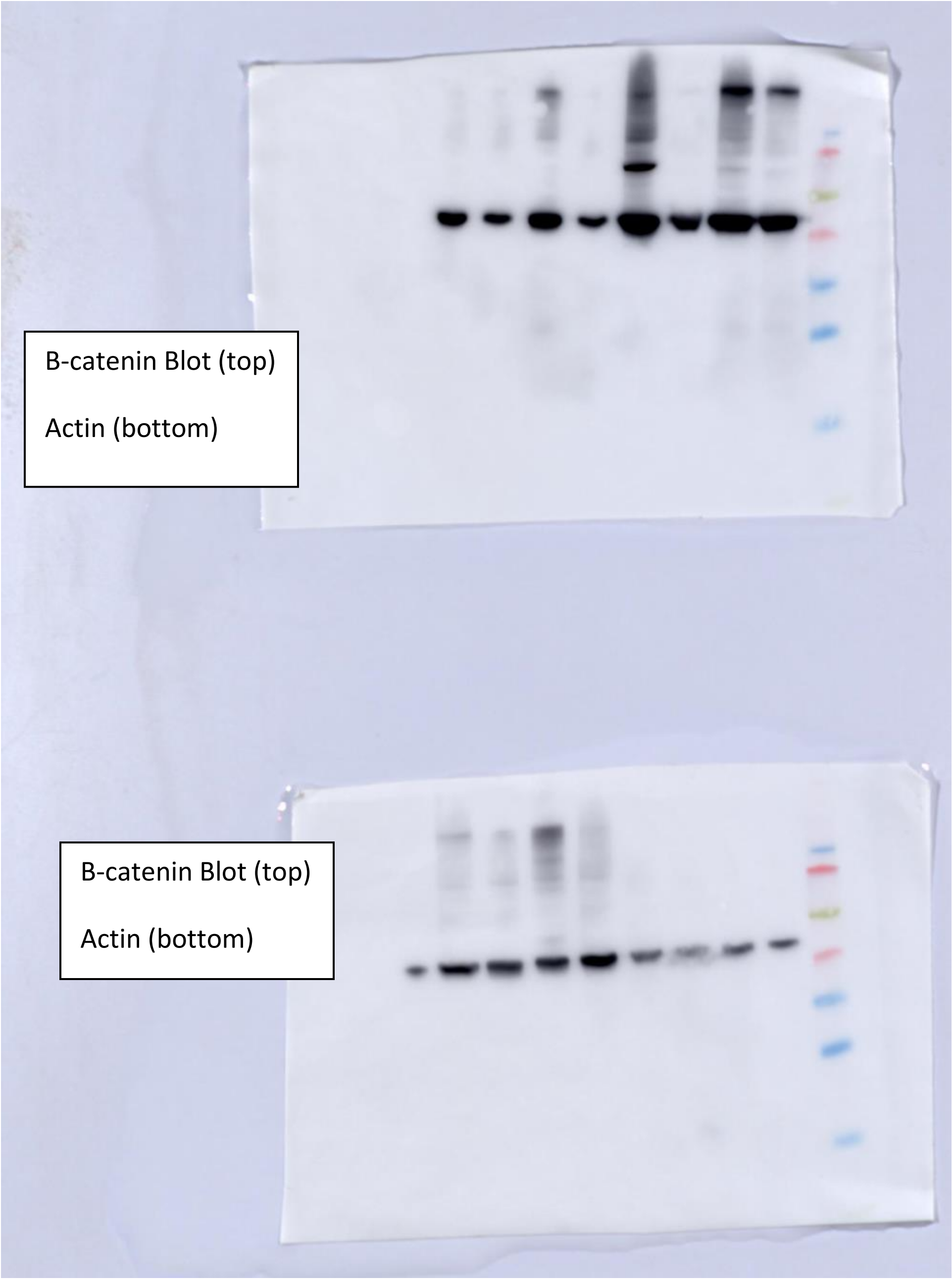
B-catenin Blot (top)
Actin (bottom)
B-catenin Blot (top)
Actin (bottom)

Table S3: Differentially expressed genes by RNAseq comparing gemcitabine (GEM) to combination therapy (Comb)

gene_id	Gem	Comb	gene_name	gene_chr	gene_start	gene_end	gene_strand	gene_length	gene_biotype	gene_description
ENSMUSG00000057003	85.022694	0.04673662	Myh4	11	67238029	67260446	+	6016	protein_coding	myosin, heavy polypeptide 4, skeletal muscle [Source:MGI Symbol;Acc:MGI:13397
ENSMUSG00000005716	113.471935	0.10803747	Pvalb	15	78191114	78206400	-	1041	protein_coding	parvalbumin [Source:MGI Symbol;Acc:MGI:97821]
ENSMUSG00000030399	37.3563199	0.13513029	Ckm	7	19404776	19422841	+	2913	protein_coding	creatine kinase, muscle [Source:MGI Symbol;Acc:MGI:88413]
ENSMUSG00000030730	46.7747322	0.29111392	Atp2a1	7	126445858	126463108	-	3477	protein_coding	ATPase, Ca++ transporting, cardiac muscle, fast twitch 1 [Source:MGI Symbol;Acc
ENSMUSG00000031972	253.825089	2.26984199	Acta1	8	123891769	123894751	-	1536	protein_coding	actin, alpha 1, skeletal muscle [Source:MGI Symbol;Acc:MGI:87902]
ENSMUSG00000006457	36.3318056	0.17487959	Actn3	19	4861223	4877909	-	2894	protein_coding	actinin alpha 3 [Source:MGI Symbol;Acc:MGI:99678]
ENSMUSG00000051985	5.31028082	0	Igfn1	1	135953578	136006342	-	8996	protein_coding	immunoglobulin-like and fibronectin type III domain containing 1 [Source:MGI Syn
ENSMUSG00000061723	212.479406	2.16692596	Tnnt3	7	142498836	142516009	+	1583	protein_coding	troponin T3, skeletal, fast [Source:MGI Symbol;Acc:MGI:109550]
ENSMUSG00000061816	62.7846096	0.46093037	Myl1	1	66924295	66945404	-	2074	protein_coding	myosin, light polypeptide 1 [Source:MGI Symbol;Acc:MGI:97269]
ENSMUSG00000019787	3.46668772	0	Trdn	10	33080554	33476709	+	9352	protein_coding	triadin [Source:MGI Symbol;Acc:MGI:1924007]
ENSMUSG00000051747	1.19224076	0.01636584	Ttn	2	76703980	76982547	-	116825	protein_coding	titin [Source:MGI Symbol;Acc:MGI:98864]
ENSMUSG00000056328	7.5194847	0.05310893	Myh1	11	67200052	67224575	+	6353	protein_coding	myosin, heavy polypeptide 1, skeletal muscle, adult [Source:MGI Symbol;Acc:MGI
ENSMUSG00000030672	106.707578	1.85326993	Mylpf	7	127208890	127214298	+	1062	protein_coding	myosin light chain, phosphorylatable, fast skeletal muscle [Source:MGI Symbol;Ac
ENSMUSG00000028834	7.77887247	0	Trim63	4	134315120	134329629	+	2842	protein_coding	tripartite motif-containing 63 [Source:MGI Symbol;Acc:MGI:2447992]
ENSMUSG00000019577	6.26289273	0	Pdk4	6	5483351	5496309	-	3511	protein_coding	pyruvate dehydrogenase kinase, isoenzyme 4 [Source:MGI Symbol;Acc:MGI:13514
ENSMUSG00000007877	60.6450405	0.88977064	Tcap	11	98383811	98384953	+	948	protein_coding	titin-cap [Source:MGI Symbol;Acc:MGI:1330233]
ENSMUSG00000031636	5.44676786	0.04370197	Pdlim3	8	45885461	45919548	+	5147	protein_coding	PDZ and LIM domain 3 [Source:MGI Symbol;Acc:MGI:1859274]
ENSMUSG00000047419	1.73057188	0.00474544	Cmya5	13	93040713	93144724	-	11850	protein_coding	cardiomyopathy associated 5 [Source:MGI Symbol;Acc:MGI:1923719]
ENSMUSG00000027559	17.3567828	0.37292726	Car3	3	14863512	14872523	+	2865	protein_coding	carbonic anhydrase 3 [Source:MGI Symbol;Acc:MGI:88270]
ENSMUSG00000061462	0.90243677	0.00972965	Obscn	11	58994256	59136402	-	28898	protein_coding	obscurin, cytoskeletal calmodulin and titin-interacting RhoGEF [Source:MGI Symb
ENSMUSG00000021798	3.83480532	0.03684423	Ldb3	14	34526603	34588682	-	6105	protein_coding	LIM domain binding 3 [Source:MGI Symbol;Acc:MGI:1344412]
ENSMUSG00000061780	28.3491187	0.36594472	Cfd	10	79890853	79892655	+	922	protein_coding	complement factor D (adipsin) [Source:MGI Symbol;Acc:MGI:87931]
ENSMUSG00000064907	136.818577	0	Gm25405	7	104423274	104423380	+	107	snRNA	predicted gene, 25405 [Source:MGI Symbol;Acc:MGI:5455182]
ENSMUSG00000079243	2.87263107	0.00963067	Xirp1	9	120013755	120023598	-	5839	protein_coding	xin actin-binding repeat containing 1 [Source:MGI Symbol;Acc:MGI:1333878]
ENSMUSG00000032648	21.8807545	0.62908831	Pygm	19	6384399	6398459	+	3218	protein_coding	muscle glycogen phosphorylase [Source:MGI Symbol;Acc:MGI:97830]
ENSMUSG00000028435	0.25924368	15.6802009	Aqp3	4	41092722	41098183	-	1829	protein_coding	aquaporin 3 [Source:MGI Symbol;Acc:MGI:1333777]
ENSMUSG00000027022	2.99836122	0.06572077	Xirp2	2	67446002	67526614	+	11979	protein_coding	xin actin-binding repeat containing 2 [Source:MGI Symbol;Acc:MGI:2685198]
ENSMUSG00000049134	4.12207217	0.06126766	Nrap	19	56320035	56390037	-	5507	protein_coding	nebulin-related anchoring protein [Source:MGI Symbol;Acc:MGI:1098765]
ENSMUSG00000068697	11.0268997	0.14860863	Myoz1	14	20649107	20656540	-	1892	protein_coding	myozenin 1 [Source:MGI Symbol;Acc:MGI:1929471]

ENSMUSG00000016349	6.79723179	0.02698345	Eef1a2	2	181147653	181157014	-		2084	protein_coding	eukaryotic translation elongation factor 1 alpha 2 [Source:MGI Symbol;Acc:MGI:1
ENSMUSG00000030785	27.6300864	0.33078532	Cox6a2	7	128205435	128206387	-		680	protein_coding	cytochrome c oxidase subunit 6A2 [Source:MGI Symbol;Acc:MGI:104649]
ENSMUSG00000031097	104.127596	4.28231098	Tnni2	7	142441808	142444410	+		1497	protein_coding	troponin I, skeletal, fast 2 [Source:MGI Symbol;Acc:MGI:105070]
ENSMUSG00000026950	1.88854636	0.06134872	Neb	2	52136647	52378474	-		26582	protein_coding	nebulin [Source:MGI Symbol;Acc:MGI:97292]
ENSMUSG00000024471	6.84189426	0.07636963	Myot	18	44334074	44355724	+		2209	protein_coding	myotilin [Source:MGI Symbol;Acc:MGI:1889800]
ENSMUSG00000030592	1.89441208	0.06952581	Ryr1	7	29003344	29125179	-		21838	protein_coding	ryanodine receptor 1, skeletal muscle [Source:MGI Symbol;Acc:MGI:99659]
ENSMUSG00000029683	6.65883	0.0569165	Lmod2	6	24597762	24605414	+		1976	protein_coding	leiomodin 2 (cardiac) [Source:MGI Symbol;Acc:MGI:2135672]
ENSMUSG00000070385	4.91129038	0.09509612	Ampd1	3	103074014	103099720	+		3548	protein_coding	adenosine monophosphate deaminase 1 [Source:MGI Symbol;Acc:MGI:88015]
ENSMUSG00000026208	29.6663697	1.52792155	Des	1	75360329	75368579	+		3754	protein_coding	desmin [Source:MGI Symbol;Acc:MGI:94885]
ENSMUSG00000031927	0.43420942	20.3237087	1700012B09	9	14756587	14771030	-		819	protein_coding	RIKEN cDNA 1700012B09 gene [Source:MGI Symbol;Acc:MGI:1916575]
ENSMUSG00000031461	1.58668156	0.02540766	Myom2	8	15057653	15133541	+		8853	protein_coding	myomesin 2 [Source:MGI Symbol;Acc:MGI:1328358]
ENSMUSG00000038403	4.17926568	0	Hfe2	3	96525172	96529210	+		2028	protein_coding	hemochromatosis type 2 (juvenile) [Source:MGI Symbol;Acc:MGI:1916835]
ENSMUSG00000006221	6.61404914	0.18277412	Hspb7	4	141420779	141425311	+		2769	protein_coding	heat shock protein family, member 7 (cardiovascular) [Source:MGI Symbol;Acc:M
ENSMUSG00000035923	6.28830128	0	Myf6	10	107492853	107494737	-		1263	protein_coding	myogenic factor 6 [Source:MGI Symbol;Acc:MGI:97253]
ENSMUSG00000076490	3.56092306	65.6182685	Trbc1	6	41538218	41539881	+		749	TR_C_gene	T cell receptor beta, constant region 1 [Source:MGI Symbol;Acc:MGI:4439726]
ENSMUSG00000040694	4.63913478	0	Apobec2	17	48419231	48432930	-		1597	protein_coding	apolipoprotein B mRNA editing enzyme, catalytic polypeptide 2 [Source:MGI Symb
ENSMUSG00000075307	5.69494242	0.15564829	Klhl41	2	69670120	69684230	+		2529	protein_coding	kelch-like 41 [Source:MGI Symbol;Acc:MGI:2683854]
ENSMUSG00000007097	3.41907529	0.15979967	Atp1a2	1	172271709	172298064	-		7038	protein_coding	ATPase, Na+/K+ transporting, alpha 2 polypeptide [Source:MGI Symbol;Acc:MGI:8
ENSMUSG00000115801	0.00572671	0.16541574	AC160336.1	14	44544055	44637201	-		93147	lincRNA	novel transcript
ENSMUSG00000038670	16.9552706	1.0900984	Mybpc2	7	44501699	44524656	-		3611	protein_coding	myosin binding protein C, fast-type [Source:MGI Symbol;Acc:MGI:1336170]
ENSMUSG00000007122	7.47325379	0.25940622	Casq1	1	172209894	172219868	-		1951	protein_coding	calsequestrin 1 [Source:MGI Symbol;Acc:MGI:1309468]
ENSMUSG00000042895	4.19801364	0.13213647	Abra	15	41864076	41869720	-		2979	protein_coding	actin-binding Rho activating protein [Source:MGI Symbol;Acc:MGI:2444891]
ENSMUSG00000040809	0.54475722	11.5430295	Chil3	3	106147554	106167564	-		1632	protein_coding	chitinase-like 3 [Source:MGI Symbol;Acc:MGI:1330860]
ENSMUSG00000020061	2.18644154	0.06210902	Mybpc1	10	88518279	88605152	-		4527	protein_coding	myosin binding protein C, slow-type [Source:MGI Symbol;Acc:MGI:1336213]
ENSMUSG00000074001	4.14721213	0.13490645	Klhl40	9	121777607	121783818	+		2501	protein_coding	kelch-like 40 [Source:MGI Symbol;Acc:MGI:1919580]
ENSMUSG00000069045	1.01461688	0	Ddx3y	Y	1260771	1286629	-		5199	protein_coding	DEAD (Asp-Glu-Ala-Asp) box polypeptide 3, Y-linked [Source:MGI Symbol;Acc:MGI
ENSMUSG00000030996	2.65858591	0.06005714	Art1	7	102101743	102113933	+		2809	protein_coding	ADP-ribosyltransferase 1 [Source:MGI Symbol;Acc:MGI:107511]
ENSMUSG00000033196	0.7058869	0	Myh2	11	67171027	67197517	+		7137	protein_coding	myosin, heavy polypeptide 2, skeletal muscle, adult [Source:MGI Symbol;Acc:MGI
ENSMUSG00000044938	0.88530179	0.0086593	Klhl31	9	77636500	77660127	+		6494	protein_coding	kelch-like 31 [Source:MGI Symbol;Acc:MGI:3045305]
ENSMUSG00000027470	5.26215911	0.36205916	Mylk2	2	152911352	152923068	+		2951	protein_coding	myosin, light polypeptide kinase 2, skeletal muscle [Source:MGI Symbol;Acc:MGI:

ENSMUSG00000020475	27.376904	2.2091734	Pgam2	11	5801640	5803733	-	840	protein_coding	phosphoglycerate mutase 2 [Source:MGI Symbol;Acc:MGI:1933118]
ENSMUSG00000038239	3.00274598	0.10957425	Hrc	7	45335290	45338974	+	2566	protein_coding	histidine rich calcium binding protein [Source:MGI Symbol;Acc:MGI:96226]
ENSMUSG00000047746	0.78424502	0	Fbxo40	16	36963460	36990467	-	5517	protein_coding	F-box protein 40 [Source:MGI Symbol;Acc:MGI:2443753]
ENSMUSG00000042717	0.71480908	0.00807373	Ppp1r3a	6	14713977	14755274	-	6965	protein_coding	protein phosphatase 1, regulatory subunit 3A [Source:MGI Symbol;Acc:MGI:21535
ENSMUSG00000024617	0.96863891	0.03766477	Camk2a	18	60925618	60988152	+	7465	protein_coding	calcium/calmodulin-dependent protein kinase II alpha [Source:MGI Symbol;Acc:M
ENSMUSG00000057000	0	1.30990119	Nxf3	X	136072099	136085255	-	3048	protein_coding	nuclear RNA export factor 3 [Source:MGI Symbol;Acc:MGI:2685230]
ENSMUSG00000020216	5.69130308	0.25311405	Jsrp1	10	80808496	80813498	-	1333	protein_coding	junctional sarcoplasmic reticulum protein 1 [Source:MGI Symbol;Acc:MGI:191670
ENSMUSG00000024028	8.01763235	77.1284885	Tff2	17	31141049	31144282	-	584	protein_coding	trefoil factor 2 (spasmolytic protein 1) [Source:MGI Symbol;Acc:MGI:1306805]
ENSMUSG00000025129	7.61049158	0.21880741	Ppp1r27	11	120549979	120551132	-	771	protein_coding	protein phosphatase 1, regulatory subunit 27 [Source:MGI Symbol;Acc:MGI:19159
ENSMUSG00000025934	0.62762453	6.33015281	Gsta3	1	21240589	21265661	+	4344	protein_coding	glutathione S-transferase, alpha 3 [Source:MGI Symbol;Acc:MGI:95856]
ENSMUSG00000005628	6.76577685	0.52401635	Tmod4	3	95124476	95129209	+	1717	protein_coding	tropomodulin 4 [Source:MGI Symbol;Acc:MGI:1355285]
ENSMUSG00000025537	1.40229163	0.08639346	Phkg1	5	129863421	129898549	-	6509	protein_coding	phosphorylase kinase gamma 1 [Source:MGI Symbol;Acc:MGI:97579]
ENSMUSG00000021622	3.31048158	0.07568439	Ckmt2	13	91853387	91876885	-	1486	protein_coding	creatine kinase, mitochondrial 2 [Source:MGI Symbol;Acc:MGI:1923972]
ENSMUSG00000027887	0.82600977	0	Sypl2	3	108211472	108226648	-	4377	protein_coding	synaptophysin-like 2 [Source:MGI Symbol;Acc:MGI:1328311]
ENSMUSG00000026580	0.37605901	4.03943372	Selp	1	164115264	164150026	+	4413	protein_coding	selectin, platelet [Source:MGI Symbol;Acc:MGI:98280]
ENSMUSG00000038132	1.00169166	0	Rbm24	13	46418434	46431095	+	3491	protein_coding	RNA binding motif protein 24 [Source:MGI Symbol;Acc:MGI:3610364]
ENSMUSG00000031382	2.51900945	0.13826292	Asb11	X	164436994	164459170	+	2847	protein_coding	ankyrin repeat and SOCS box-containing 11 [Source:MGI Symbol;Acc:MGI:191610
ENSMUSG00000052374	1.23380693	0.02963558	Actn2	13	12269426	12340760	-	3795	protein_coding	actinin alpha 2 [Source:MGI Symbol;Acc:MGI:109192]
ENSMUSG00000040666	4.75666741	0.28654015	Sh3bgr	16	96200450	96228935	+	1570	protein_coding	SH3-binding domain glutamic acid-rich protein [Source:MGI Symbol;Acc:MGI:1354
ENSMUSG00000041476	1.40683852	0	Smpx	X	157698910	157752591	+	2275	protein_coding	small muscle protein, X-linked [Source:MGI Symbol;Acc:MGI:1913356]
ENSMUSG00000004654	0	0.8869638	Ghrhr	6	55376295	55388530	+	3487	protein_coding	growth hormone releasing hormone receptor [Source:MGI Symbol;Acc:MGI:95710
ENSMUSG00000044951	0.7862528	0.043881	Mylk4	13	32700834	32783954	-	7689	protein_coding	myosin light chain kinase family, member 4 [Source:MGI Symbol;Acc:MGI:364375
ENSMUSG00000043102	0.60747782	5.57147809	Qrfp	2	31806166	31810580	-	2927	protein_coding	pyroglutamylated RFamide peptide [Source:MGI Symbol;Acc:MGI:3630329]
ENSMUSG00000079588	1.30934284	0.02070453	Tmem182	1	40805601	40856887	+	2716	protein_coding	transmembrane protein 182 [Source:MGI Symbol;Acc:MGI:1923725]
ENSMUSG00000000031	7.73395817	0.98294338	H19	7	142575529	142578143	-	2460	lincRNA	H19, imprinted maternally expressed transcript [Source:MGI Symbol;Acc:MGI:958
ENSMUSG00000029685	0.63263067	0	Asb15	6	24528144	24573164	+	4497	protein_coding	ankyrin repeat and SOCS box-containing 15 [Source:MGI Symbol;Acc:MGI:192616
ENSMUSG00000026407	0.50673234	0.00858527	Cacna1s	1	136052750	136119822	+	6550	protein_coding	calcium channel, voltage-dependent, L type, alpha 1S subunit [Source:MGI Symbo
ENSMUSG00000020722	4.41318775	0.27013693	Cacng1	11	107703218	107716522	-	1249	protein_coding	calcium channel, voltage-dependent, gamma subunit 1 [Source:MGI Symbol;Acc:M
ENSMUSG00000025172	3.87946381	0.1533641	Ankrd2	19	42036038	42045110	+	1100	protein_coding	ankyrin repeat domain 2 (stretch responsive muscle) [Source:MGI Symbol;Acc:MG
ENSMUSG00000109724	0.12053664	1.71543231	Gm18194	7	12795723	12799164	-	3442	transcribed_process	predicted gene, 18194 [Source:MGI Symbol;Acc:MGI:5010379]

ENSMUSG00000054863	0.65221002	5.35649271	Fam19a5	15	87544299	87759364	+	2908	protein_coding	family with sequence similarity 19, member A5 [Source:MGI Symbol;Acc:MGI:214
ENSMUSG00000063388	3.04318168	0.09166015	BC023105	18	60441753	60442983	+	1227	pseudogene	cDNA sequence BC023105 [Source:MGI Symbol;Acc:MGI:2384767]
ENSMUSG00000021795	0.90731265	7.11934317	Sftpd	14	41172214	41185149	-	2417	protein_coding	surfactant associated protein D [Source:MGI Symbol;Acc:MGI:109515]
ENSMUSG00000002500	3.24666022	0.20535668	Rpl3l	17	24727820	24736143	+	1643	protein_coding	ribosomal protein L3-like [Source:MGI Symbol;Acc:MGI:1913461]
ENSMUSG00000062077	2.55899327	0.07831964	Trim54	5	31116712	31137630	+	1436	protein_coding	tripartite motif-containing 54 [Source:MGI Symbol;Acc:MGI:1889623]
ENSMUSG00000097767	0.15306898	1.3070523	Miat	5	112213228	112229395	-	9293	lincRNA	myocardial infarction associated transcript (non-protein coding) [Source:MGI Sym
ENSMUSG00000071540	0.46802975	0	3425401B19R	14	32659119	32685293	-	5572	protein_coding	RIKEN cDNA 3425401B19 gene [Source:MGI Symbol;Acc:MGI:3588196]
ENSMUSG00000031397	2.08510068	0.1364339	Tktl1	X	74177259	74208500	+	2473	protein_coding	transketolase-like 1 [Source:MGI Symbol;Acc:MGI:1933244]
ENSMUSG00000018566	2.31761463	0.24252518	Slc2a4	11	69942539	69948188	-	3478	protein_coding	solute carrier family 2 (facilitated glucose transporter), member 4 [Source:MGI Sy
ENSMUSG00000095918	0.05407809	2.77062888	Gm5861	5	11183072	11187810	+	1096	protein_coding	predicted gene 5861 [Source:MGI Symbol;Acc:MGI:3644254]
ENSMUSG00000044086	1.15060596	0	Lmod3	6	97238534	97252759	-	2215	protein_coding	leiomodin 3 (fetal) [Source:MGI Symbol;Acc:MGI:2444169]
ENSMUSG00000078815	1.22916187	0	Cacng6	7	3424195	3435667	+	1977	protein_coding	calcium channel, voltage-dependent, gamma subunit 6 [Source:MGI Symbol;Acc:M
ENSMUSG00000002831	4.53473377	0.71058436	Plin4	17	56100591	56109803	-	5777	protein_coding	perilipin 4 [Source:MGI Symbol;Acc:MGI:1929709]
ENSMUSG00000022215	2.44158953	0	Fitm1	14	55575617	55576954	+	971	protein_coding	fat storage-inducing transmembrane protein 1 [Source:MGI Symbol;Acc:MGI:1915
ENSMUSG00000079278	0.9331909	0	Tmem233	5	116038755	116083244	-	2477	protein_coding	transmembrane protein 233 [Source:MGI Symbol;Acc:MGI:3651514]
ENSMUSG00000056423	3.26515084	21.7526012	Uts2b	16	27353322	27370239	-	835	protein_coding	urotensin 2B [Source:MGI Symbol;Acc:MGI:2677064]
ENSMUSG00000010796	0.02095072	0.95412097	Asz1	6	18050964	18109061	-	2829	protein_coding	ankyrin repeat, SAM and basic leucine zipper domain containing 1 [Source:MGI Sy
ENSMUSG00000027513	0.79510089	0.01639939	Pck1	2	173153048	173159273	+	3429	protein_coding	phosphoenolpyruvate carboxykinase 1, cytosolic [Source:MGI Symbol;Acc:MGI:97
ENSMUSG00000039376	0.58898271	0.01214809	Synpo2l	14	20658946	20668354	-	4629	protein_coding	synaptopodin 2-like [Source:MGI Symbol;Acc:MGI:1916010]
ENSMUSG00000022949	0.18171564	1.76717452	Clic6	16	92485736	92541243	+	3914	protein_coding	chloride intracellular channel 6 [Source:MGI Symbol;Acc:MGI:2146607]
ENSMUSG00000025488	9.54898885	0.46861254	Cox8b	7	140898945	140900446	-	360	protein_coding	cytochrome c oxidase subunit 8B [Source:MGI Symbol;Acc:MGI:105958]
ENSMUSG00000049641	1.32907556	0	Vgll2	10	52022502	52028471	+	1650	protein_coding	vestigial like family member 2 [Source:MGI Symbol;Acc:MGI:2447460]
ENSMUSG00000056673	0.14461716	0	Kdm5d	Y	897788	956786	+	15164	protein_coding	lysine (K)-specific demethylase 5D [Source:MGI Symbol;Acc:MGI:99780]
ENSMUSG00000031519	5.33844345	0.73228655	Asb5	8	54529580	54587842	+	1843	protein_coding	ankyrin repeat and SOCs box-containing 5 [Source:MGI Symbol;Acc:MGI:1923544]
ENSMUSG00000043441	0.06911099	0.9442193	Gpr149	3	62529077	62605140	-	4288	protein_coding	G protein-coupled receptor 149 [Source:MGI Symbol;Acc:MGI:2443628]
ENSMUSG00000038763	0.32186742	0	Alpk3	7	81057600	81105612	+	6445	protein_coding	alpha-kinase 3 [Source:MGI Symbol;Acc:MGI:2151224]
ENSMUSG00000022032	0.17509479	1.35669214	Scara5	14	65666403	65764826	+	6093	protein_coding	scavenger receptor class A, member 5 [Source:MGI Symbol;Acc:MGI:1918395]
ENSMUSG00000029121	1.89265638	0.30327465	Crmp1	5	37241940	37292133	+	7046	protein_coding	collapsin response mediator protein 1 [Source:MGI Symbol;Acc:MGI:107793]
ENSMUSG00000035681	0.48989991	0.03151219	Kcnc2	10	112271121	112467024	+	7138	protein_coding	potassium voltage gated channel, Shaw-related subfamily, member 2 [Source:MG
ENSMUSG00000037621	0.42554948	3.20757526	Atoh8	6	72206177	72235577	-	2507	protein_coding	atonal bHLH transcription factor 8 [Source:MGI Symbol;Acc:MGI:1918343]

ENSMUSG00000056468	3.56960536	0.57004745	5730596B20R	6	52177498	52180851	+	3354	protein_coding	RIKEN cDNA 5730596B20 gene [Source:MGI Symbol;Acc:MGI:1924830]
ENSMUSG00000059654	34.3553795	171.561954	Reg1	6	78422711	78428667	+	904	protein_coding	regenerating islet-derived 1 [Source:MGI Symbol;Acc:MGI:97895]
ENSMUSG00000032942	0.96419422	0.05381197	Ucp3	7	100472990	100486432	+	3135	protein_coding	uncoupling protein 3 (mitochondrial, proton carrier) [Source:MGI Symbol;Acc:MGI
ENSMUSG00000001027	0.42930676	0.02395974	Scn4a	11	106318592	106353288	-	7041	protein_coding	sodium channel, voltage-gated, type IV, alpha [Source:MGI Symbol;Acc:MGI:9825
ENSMUSG00000028341	0.60388254	0.04696301	Nr4a3	4	48045153	48086447	+	5987	protein_coding	nuclear receptor subfamily 4, group A, member 3 [Source:MGI Symbol;Acc:MGI:13
ENSMUSG00000051910	0.12387791	0	Sox6	7	115470872	116038796	-	14832	protein_coding	SRY (sex determining region Y)-box 6 [Source:MGI Symbol;Acc:MGI:98368]
ENSMUSG00000055489	0.33348616	0.01438197	Ano5	7	51511029	51598709	+	7820	protein_coding	anoctamin 5 [Source:MGI Symbol;Acc:MGI:3576659]
ENSMUSG00000030800	0.28802715	2.55054773	Prss8	7	127925716	127930104	-	1852	protein_coding	protease, serine 8 (prostasin) [Source:MGI Symbol;Acc:MGI:1923810]
ENSMUSG00000094036	0.05397959	1.99736492	Gm6465	5	11845539	11850352	+	1098	protein_coding	predicted gene 6465 [Source:MGI Symbol;Acc:MGI:3642949]
ENSMUSG00000068457	0.18160429	0	Uty	Y	1096861	1245759	-	9791	protein_coding	ubiquitously transcribed tetratricopeptide repeat gene, Y chromosome [Source:M
ENSMUSG00000028464	36.7557006	7.98364986	Tpm2	4	43514711	43523765	-	2909	protein_coding	tropomyosin 2, beta [Source:MGI Symbol;Acc:MGI:98810]
ENSMUSG00000026535	0.41911316	3.34683794	Ifi202b	1	173962568	173982744	-	1697	protein_coding	interferon activated gene 202B [Source:MGI Symbol;Acc:MGI:1347083]
ENSMUSG00000052912	0.66325928	0.01748011	Smarca5-ps	4	145464204	145467420	+	3217	transcribed_process	SWI/SNF related, matrix associated, actin depenent ragulator of chromatin, subfa
ENSMUSG00000028396	0.79648656	0	2310002L09R	4	73939371	73950846	-	2158	protein_coding	RIKEN cDNA 2310002L09 gene [Source:MGI Symbol;Acc:MGI:1916780]
ENSMUSG00000020067	0.77383414	0.08694409	Mypn	10	63115795	63203952	-	5821	protein_coding	myopalladin [Source:MGI Symbol;Acc:MGI:1916052]
ENSMUSG00000044676	0.19486959	1.25723436	Zfp612	8	110079746	110092741	+	6083	protein_coding	zinc finger protein 612 [Source:MGI Symbol;Acc:MGI:2443465]
ENSMUSG00000101451	0.82359722	0	Gm29430	6	52183110	52189925	+	2015	antisense	predicted gene 29430 [Source:MGI Symbol;Acc:MGI:5580136]
ENSMUSG00000036523	0.15467011	0.92744194	Greb1	12	16670615	16800886	-	9580	protein_coding	gene regulated by estrogen in breast cancer protein [Source:MGI Symbol;Acc:MG
ENSMUSG00000046093	0.7437839	3.68640694	Hpcal4	4	123183227	123194701	+	5339	protein_coding	hippocalcin-like 4 [Source:MGI Symbol;Acc:MGI:2157521]
ENSMUSG00000027792	0.38686571	2.05213502	Bche	3	73635808	73708415	-	6741	protein_coding	butyrylcholinesterase [Source:MGI Symbol;Acc:MGI:894278]
ENSMUSG00000087090	0.58614483	0.01635646	Nctc1	7	142544609	142558596	-	3438	processed_transcrip	non-coding transcript 1 [Source:MGI Symbol;Acc:MGI:1306816]
ENSMUSG00000032845	0.25895219	0.0072261	Alpk2	18	65265529	65393888	-	7782	protein_coding	alpha-kinase 2 [Source:MGI Symbol;Acc:MGI:2449492]
ENSMUSG00000032523	1.85146897	0.23564516	Hhatl	9	121784016	121792507	-	2625	protein_coding	hedgehog acyltransferase-like [Source:MGI Symbol;Acc:MGI:1922020]
ENSMUSG00000055030	0	2.52051258	Sprr2e	3	92352143	92353449	+	647	protein_coding	small proline-rich protein 2E [Source:MGI Symbol;Acc:MGI:1330346]
ENSMUSG00000035296	0.60479169	0.02942622	Sgcg	14	61219115	61258490	-	3822	protein_coding	sarcoglycan, gamma (dystrophin-associated glycoprotein) [Source:MGI Symbol;Ac
ENSMUSG00000026527	0.4332716	0.028028	Rgs7	1	175059087	175492500	-	6019	protein_coding	regulator of G protein signaling 7 [Source:MGI Symbol;Acc:MGI:1346089]
ENSMUSG00000021850	0.57440558	2.99739926	ccdc198	14	49219588	49245474	-	3921	protein_coding	coiled-coil domain containing 198 [Source:MGI Symbol;Acc:MGI:1914332]
ENSMUSG00000023032	0.21848861	1.10990022	Slc4a8	15	100761747	100823968	+	13021	protein_coding	solute carrier family 4 (anion exchanger), member 8 [Source:MGI Symbol;Acc:MGI
ENSMUSG00000021373	1.01181549	0.12168828	Cap2	13	46501848	46650281	+	4159	protein_coding	CAP, adenylate cyclase-associated protein, 2 (yeast) [Source:MGI Symbol;Acc:MG
ENSMUSG00000069917	0.08885995	2.8664755	Hba-a2	11	32296489	32297298	+	667	protein_coding	hemoglobin alpha, adult chain 2 [Source:MGI Symbol;Acc:MGI:96016]

ENSMUSG00000039891	0.96916111	0.15325265	Txlnb	10	17796226	17845665	+	5504	protein_coding	taxilin beta [Source:MGI Symbol;Acc:MGI:2671945]
ENSMUSG00000056900	0.60837317	0.08140131	Usp13	3	32817546	32938071	+	7599	protein_coding	ubiquitin specific peptidase 13 (isopeptidase T-3) [Source:MGI Symbol;Acc:MGI:1
ENSMUSG00000035429	0.07077811	0.72524702	Ptprh	7	4548612	4604041	-	4187	protein_coding	protein tyrosine phosphatase, receptor type, H [Source:MGI Symbol;Acc:MGI:302
ENSMUSG00000027656	8.31745749	1.78287099	Wisp2	2	163820861	163833146	+	1924	protein_coding	WNT1 inducible signaling pathway protein 2 [Source:MGI Symbol;Acc:MGI:13283
ENSMUSG00000029074	0.28259025	1.70746652	Ttll10	4	156034840	156059414	-	3985	protein_coding	tubulin tyrosine ligase-like family, member 10 [Source:MGI Symbol;Acc:MGI:1921
ENSMUSG00000069355	0	1.76958581	Gm5152	5	10242041	10246526	-	858	protein_coding	predicted gene 5152 [Source:MGI Symbol;Acc:MGI:3643407]
ENSMUSG00000027895	0.40407408	0	Kcnc4	3	107438303	107459552	-	3667	protein_coding	potassium voltage gated channel, Shaw-related subfamily, member 4 [Source:MG
ENSMUSG00000037600	0.79723697	4.13655457	Kdf1	4	133518963	133530790	+	2379	protein_coding	keratinocyte differentiation factor 1 [Source:MGI Symbol;Acc:MGI:1916323]
ENSMUSG00000072720	0.37673695	0.04730805	Myo18b	5	112688876	112896362	-	10698	protein_coding	myosin XVIIIb [Source:MGI Symbol;Acc:MGI:1921626]
ENSMUSG00000079560	7.53928056	1.81398402	Hoxa3	6	52169062	52213336	-	6014	protein_coding	homeobox A3 [Source:MGI Symbol;Acc:MGI:96175]
ENSMUSG00000034472	1.00773225	0.10623458	Rasd2	8	75213944	75224113	+	3176	protein_coding	RASD family, member 2 [Source:MGI Symbol;Acc:MGI:1922391]
ENSMUSG00000028392	0.85678823	4.32749335	Bspry	4	62480053	62497298	+	2352	protein_coding	B-box and SPRY domain containing [Source:MGI Symbol;Acc:MGI:2177191]
ENSMUSG00000020396	0.02967931	0.53502083	Nefh	11	4938754	4948064	-	3994	protein_coding	neurofilament, heavy polypeptide [Source:MGI Symbol;Acc:MGI:97309]
ENSMUSG00000022269	0.0416219	0.75030659	11-Mar	15	26309048	26409576	+	2848	protein_coding	membrane-associated ring finger (C3HC4) 11 [Source:MGI Symbol;Acc:MGI:3608
ENSMUSG00000103472	0.02272171	0.40959808	Pcdhga7	18	37714764	37841873	+	5217	protein_coding	protocadherin gamma subfamily A, 7 [Source:MGI Symbol;Acc:MGI:1935219]
ENSMUSG00000091721	2.20079128	10.2242736	Gimd1	3	132629820	132645351	+	1562	protein_coding	GIMAP family P-loop NTPase domain containing 1 [Source:MGI Symbol;Acc:MGI:3
ENSMUSG00000045871	4.01187999	0.90185809	Slitrk6	14	110748580	110755149	-	4240	protein_coding	SLIT and NTRK-like family, member 6 [Source:MGI Symbol;Acc:MGI:2443198]
ENSMUSG00000022464	0.91804569	0.1187753	Slc38a4	15	96994820	97055956	-	4261	protein_coding	solute carrier family 38, member 4 [Source:MGI Symbol;Acc:MGI:1916604]
ENSMUSG00000039323	0	0.79159238	Igfbp2	1	72824503	72852474	+	1847	protein_coding	insulin-like growth factor binding protein 2 [Source:MGI Symbol;Acc:MGI:96437]
ENSMUSG00000019893	0	0.18802355	Ros1	10	52045721	52195244	-	7776	protein_coding	Ros1 proto-oncogene [Source:MGI Symbol;Acc:MGI:97999]
ENSMUSG00000034607	0.36604994	1.98249875	Pof1b	X	112638431	112698651	-	3886	protein_coding	premature ovarian failure 1B [Source:MGI Symbol;Acc:MGI:1916943]
ENSMUSG00000035540	1.38135078	6.64439243	Gc	5	89417522	89457898	-	1845	protein_coding	vitamin D binding protein [Source:MGI Symbol;Acc:MGI:95669]
ENSMUSG00000030351	0.17805358	1.08750488	Tspan11	6	127887589	127953977	+	5326	protein_coding	tetraspanin 11 [Source:MGI Symbol;Acc:MGI:1915748]
ENSMUSG00000028328	1.75735975	0.32776751	Tmod1	4	46038935	46116032	+	3946	protein_coding	tropomodulin 1 [Source:MGI Symbol;Acc:MGI:98775]
ENSMUSG00000027386	14.4134526	3.64089296	Fbln7	2	128862981	128897034	+	3228	protein_coding	fibulin 7 [Source:MGI Symbol;Acc:MGI:1917620]
ENSMUSG00000006542	0.4668164	0.01527254	Prkag3	1	74738922	74749221	-	3682	protein_coding	protein kinase, AMP-activated, gamma 3 non-catalytic subunit [Source:MGI Symb
ENSMUSG00000097580	2.41337236	0.33672757	Gm5432	12	15817003	15888155	+	1670	bidirectional_promo	predicted gene 5432 [Source:MGI Symbol;Acc:MGI:3649057]
ENSMUSG00000041144	0.08602693	0.43343142	Dnah7b	1	46066315	46373546	+	19980	protein_coding	dynein, axonemal, heavy chain 7B [Source:MGI Symbol;Acc:MGI:2684953]
ENSMUSG00000053093	0.15821733	0	Myh7	14	54970684	54994626	-	8616	protein_coding	myosin, heavy polypeptide 7, cardiac muscle, beta [Source:MGI Symbol;Acc:MGI:2
ENSMUSG00000029120	0.02663802	0.45492273	Ppp2r2c	5	36868513	36955078	+	4450	protein_coding	protein phosphatase 2, regulatory subunit B, gamma [Source:MGI Symbol;Acc:MG

ENSMUSG00000091712	0.3510742	0.01959356	Sec14l5	16	5147109	5187271	+	5740	protein_coding	SEC14-like lipid binding 5 [Source:MGI Symbol;Acc:MGI:3616084]
ENSMUSG00000059430	2.36012481	10.2571068	Actg2	6	83512905	83536265	-	1557	protein_coding	actin, gamma 2, smooth muscle, enteric [Source:MGI Symbol;Acc:MGI:104589]
ENSMUSG00000060808	2.90192335	0.166865	B9d1os	11	61504386	61505059	-	674	antisense	B9 protein domain 1, opposite strand [Source:MGI Symbol;Acc:MGI:1920466]
ENSMUSG00000032355	0.14624617	0	Mlip	9	77102081	77352969	-	8916	protein_coding	muscular LMNA-interacting protein [Source:MGI Symbol;Acc:MGI:1916892]
ENSMUSG00000073375	0.74086982	0	Lrrc30	17	67630964	67632723	-	1760	protein_coding	leucine rich repeat containing 30 [Source:MGI Symbol;Acc:MGI:2685172]
ENSMUSG00000028078	0.64588291	0.12788819	Dclk2	3	86786151	86920852	-	10553	protein_coding	doublecortin-like kinase 2 [Source:MGI Symbol;Acc:MGI:1918012]
ENSMUSG00000018569	12.8397152	49.1321484	Cldn7	11	69964779	69967885	+	1948	protein_coding	claudin 7 [Source:MGI Symbol;Acc:MGI:1859285]
ENSMUSG00000016529	3.98146837	0.72974777	Il10	1	131019845	131024974	+	1310	protein_coding	interleukin 10 [Source:MGI Symbol;Acc:MGI:96537]
ENSMUSG00000058427	3.43924096	13.8136462	Cxcl2	5	90903871	90905938	+	2068	protein_coding	chemokine (C-X-C motif) ligand 2 [Source:MGI Symbol;Acc:MGI:1340094]
ENSMUSG00000026051	0.39425445	3.24184284	1500015O10	1	43730602	43742578	+	902	protein_coding	RIKEN cDNA 1500015O10 gene [Source:MGI Symbol;Acc:MGI:1926146]
ENSMUSG00000021898	1.60439444	0.26473207	Asb14	14	26894557	26915258	+	2549	protein_coding	ankyrin repeat and SOCS box-containing 14 [Source:MGI Symbol;Acc:MGI:265510
ENSMUSG00000043795	5.14948703	1.19747668	Prr33	7	142491074	142506771	-	2348	lincRNA	proline rich 33 [Source:MGI Symbol;Acc:MGI:3642289]
ENSMUSG00000030849	0.33284624	1.42108295	Fgfr2	7	130162451	133123350	-	10328	protein_coding	fibroblast growth factor receptor 2 [Source:MGI Symbol;Acc:MGI:95523]
ENSMUSG00000018893	1.27119755	0.07538003	Mb	15	77015487	77050670	-	1492	protein_coding	myoglobin [Source:MGI Symbol;Acc:MGI:96922]
ENSMUSG00000042379	3.92869454	15.4192967	Esm1	13	113209659	113218098	+	2097	protein_coding	endothelial cell-specific molecule 1 [Source:MGI Symbol;Acc:MGI:1918940]
ENSMUSG00000026417	2.54748779	0.57281334	Pigr	1	130826684	130852249	+	4025	protein_coding	polymeric immunoglobulin receptor [Source:MGI Symbol;Acc:MGI:103080]
ENSMUSG00000030017	2.91581691	11.5446268	Reg3g	6	78466269	78468872	-	2114	protein_coding	regenerating islet-derived 3 gamma [Source:MGI Symbol;Acc:MGI:109406]
ENSMUSG00000018845	1.89958166	0.41302151	Unc45b	11	82910550	82943403	+	4493	protein_coding	unc-45 myosin chaperone B [Source:MGI Symbol;Acc:MGI:2443377]
ENSMUSG00000096045	0	0.43169914	Gm21698	5	25982030	25988650	-	2996	protein_coding	predicted gene, 21698 [Source:MGI Symbol;Acc:MGI:5435053]
ENSMUSG00000068614	0.76925915	0	Actc1	2	114047282	114053548	-	1618	protein_coding	actin, alpha, cardiac muscle 1 [Source:MGI Symbol;Acc:MGI:87905]
ENSMUSG00000053101	0.1587401	0.89423886	Gpr141	13	19749682	19824257	-	5974	protein_coding	G protein-coupled receptor 141 [Source:MGI Symbol;Acc:MGI:2672983]
ENSMUSG00000075014	19.7565287	4.77132767	Gm10800	2	98666547	98667301	-	660	protein_coding	predicted gene 10800 [Source:MGI Symbol;Acc:MGI:3641657]
ENSMUSG00000033208	3.95396834	15.5362109	S100b	10	76253853	76261159	+	1484	protein_coding	S100 protein, beta polypeptide, neural [Source:MGI Symbol;Acc:MGI:98217]
ENSMUSG00000049670	0.16318719	1.42441697	Morn4	19	42074939	42086370	-	1816	protein_coding	MORN repeat containing 4 [Source:MGI Symbol;Acc:MGI:2449568]
ENSMUSG00000040254	2.77212025	0.72622676	Sema3d	5	12383385	12588948	+	8595	protein_coding	sema domain, immunoglobulin domain (Ig), short basic domain, secreted, (semap
ENSMUSG00000055027	0.86049113	0.13606875	Smyd1	6	71213940	71322233	-	4546	protein_coding	SET and MYND domain containing 1 [Source:MGI Symbol;Acc:MGI:104790]
ENSMUSG00000043003	0.51574648	2.1824002	Rasef	4	73714579	73790994	-	5746	protein_coding	RAS and EF hand domain containing [Source:MGI Symbol;Acc:MGI:2448565]
ENSMUSG00000029158	0.97242963	0	Yipf7	5	69516671	69542648	-	1219	protein_coding	Yip1 domain family, member 7 [Source:MGI Symbol;Acc:MGI:1922831]
ENSMUSG00000069372	0.73856182	0	Ctxn3	18	57468516	57478133	+	1605	protein_coding	cortexin 3 [Source:MGI Symbol;Acc:MGI:3642816]
ENSMUSG00000026574	4.64117988	1.04377735	Dpt	1	164796644	164824266	+	1724	protein_coding	dermatopontin [Source:MGI Symbol;Acc:MGI:1928392]

ENSMUSG00000086054	1.08156179	5.13079423	Hnf1aos1	5	114968784	114998042	+	1370	antisense	HNF1 homeobox A, opposite strand 1 [Source:MGI Symbol;Acc:MGI:3652225]
ENSMUSG00000030554	1.51666784	0.3756934	Synm	7	67730160	67759742	-	7933	protein_coding	synemin, intermediate filament protein [Source:MGI Symbol;Acc:MGI:2661187]
ENSMUSG00000026579	0.32705963	1.37745597	F5	1	164151838	164220277	+	7430	protein_coding	coagulation factor V [Source:MGI Symbol;Acc:MGI:88382]
ENSMUSG00000042784	17.9375132	63.6641895	Muc1	3	89229057	89233381	+	2346	protein_coding	mucin 1, transmembrane [Source:MGI Symbol;Acc:MGI:97231]
ENSMUSG00000030616	1.16915115	4.21519349	Sytl2	7	90302252	90410719	+	13941	protein_coding	synaptotagmin-like 2 [Source:MGI Symbol;Acc:MGI:1933366]
ENSMUSG00000054892	0.15689804	0.70323963	Txk	5	72695978	72752777	-	10955	protein_coding	TXK tyrosine kinase [Source:MGI Symbol;Acc:MGI:102960]
ENSMUSG00000069919	2.55472353	10.9419874	Hba-a1	11	32283511	32284465	+	812	protein_coding	hemoglobin alpha, adult chain 1 [Source:MGI Symbol;Acc:MGI:96015]
ENSMUSG00000051596	0.03443904	0.50646097	Otop1	5	38275972	38304217	+	3442	protein_coding	otopetrin 1 [Source:MGI Symbol;Acc:MGI:2388363]
ENSMUSG00000079440	0	0.50530749	Alpi	1	87098002	87101606	-	2337	protein_coding	alkaline phosphatase, intestinal [Source:MGI Symbol;Acc:MGI:1924018]
ENSMUSG00000112929	0	2.21557899	Gm47729	10	16584268	16644555	+	533	lincRNA	predicted gene, 47729 [Source:MGI Symbol;Acc:MGI:6096862]
ENSMUSG00000083773	2.43005302	0.28116752	Gm13394	2	28933610	28934609	-	1000	processed_pseudog	predicted gene 13394 [Source:MGI Symbol;Acc:MGI:3651848]
ENSMUSG00000031284	0.17994326	0.78887036	Pak3	X	143518591	143797796	+	9552	protein_coding	p21 (RAC1) activated kinase 3 [Source:MGI Symbol;Acc:MGI:1339656]
ENSMUSG00000022126	0.18076885	1.17912419	Acod1	14	103046977	103056573	+	2623	protein_coding	aconitate decarboxylase 1 [Source:MGI Symbol;Acc:MGI:103206]
ENSMUSG00000020159	12.5840371	43.0618428	Gabrp	11	33550781	33578959	-	3377	protein_coding	gamma-aminobutyric acid (GABA) A receptor, pi [Source:MGI Symbol;Acc:MGI:238
ENSMUSG00000030607	5.35640253	18.3479784	Acan	7	79053483	79115099	+	7469	protein_coding	aggrecan [Source:MGI Symbol;Acc:MGI:99602]
ENSMUSG00000021214	0.93299265	4.56576923	Akr1c18	13	4132615	4150654	-	1207	protein_coding	aldo-keto reductase family 1, member C18 [Source:MGI Symbol;Acc:MGI:2145420
ENSMUSG00000037705	0.75976395	0.16693255	Tecta	9	42329619	42399929	-	7411	protein_coding	tectorin alpha [Source:MGI Symbol;Acc:MGI:109575]
ENSMUSG00000084939	0.50773489	2.18382543	Gm830	4	95464724	95487394	-	3502	processed_transcrip	predicted gene 830 [Source:MGI Symbol;Acc:MGI:2685676]
ENSMUSG00000052305	5.17065398	19.5499294	Hbb-bs	7	103826534	103828096	-	768	protein_coding	hemoglobin, beta adult s chain [Source:MGI Symbol;Acc:MGI:5474852]
ENSMUSG00000097084	0.87644489	0.10394363	Foxl1	8	121127940	121130644	+	2705	protein_coding	forkhead box L1 [Source:MGI Symbol;Acc:MGI:1347469]
ENSMUSG00000024049	1.49604845	0.40324246	Myom1	17	71002633	71126856	+	10459	protein_coding	myomesin 1 [Source:MGI Symbol;Acc:MGI:1341430]
ENSMUSG00000022206	0.3345711	1.30566663	Npr3	15	11839896	11907287	-	9389	protein_coding	natriuretic peptide receptor 3 [Source:MGI Symbol;Acc:MGI:97373]
ENSMUSG00000017639	0.27045577	1.11697204	Rab11fip4	11	79591212	79698023	+	7451	protein_coding	RAB11 family interacting protein 4 (class II) [Source:MGI Symbol;Acc:MGI:244292
ENSMUSG00000052736	0.35692283	1.46098748	Klrc2	6	129647496	129660689	-	5812	protein_coding	killer cell lectin-like receptor subfamily C, member 2 [Source:MGI Symbol;Acc:MG
ENSMUSG00000112329	3.14559255	0	Gm47908	10	33477115	33477472	-	358	TEC	predicted gene, 47908 [Source:MGI Symbol;Acc:MGI:6097152]
ENSMUSG00000111171	0	0.63371091	Gm47815	10	43034993	43050219	+	1686	antisense	predicted gene, 47815 [Source:MGI Symbol;Acc:MGI:6097001]
ENSMUSG00000071317	0.24427812	0	Bves	10	45335772	45372479	+	4610	protein_coding	blood vessel epicardial substance [Source:MGI Symbol;Acc:MGI:1346013]
ENSMUSG00000019874	0	0.85474927	Fabp7	10	57784881	57788450	+	1250	protein_coding	fatty acid binding protein 7, brain [Source:MGI Symbol;Acc:MGI:101916]
ENSMUSG00000025370	0	0.30190353	Cdh9	15	16728756	16857094	+	3539	protein_coding	cadherin 9 [Source:MGI Symbol;Acc:MGI:107433]
ENSMUSG00000042485	21.0512526	6.05101628	Mustn1	14	30879200	30881610	+	1236	protein_coding	musculoskeletal, embryonic nuclear protein 1 [Source:MGI Symbol;Acc:MGI:19134
ENSMUSG00000060402	0.10928016	0.60324242	Chst8	7	34674468	34812711	-	5966	protein_coding	carbohydrate (N-acetylgalactosamine 4-0) sulfotransferase 8 [Source:MGI Symbo
ENSMUSG00000099068	6.37009569	0.26277339	Gm27861	6	52161042	52161255	+	214	misc_RNA	predicted gene, 27861 [Source:MGI Symbol;Acc:MGI:5531243]
